# Genome-wide association study of antisocial personality disorder

**DOI:** 10.1038/tp.2016.155

**Published:** 2016-09-06

**Authors:** M-R Rautiainen, T Paunio, E Repo-Tiihonen, M Virkkunen, H M Ollila, S Sulkava, O Jolanki, A Palotie, J Tiihonen

**Affiliations:** 1 National Institute for Health and Welfare, Department of Health, Helsinki, Finland; 2 Department of Forensic Psychiatry, Niuvanniemi Hospital, University of Eastern Finland, Kuopio, Finland; 3 Department of Psychiatry, University of Helsinki, Helsinki, Finland; 4 Department of Psychiatry, Helsinki University Hospital, Helsinki, Finland; 5 Institute for Molecular Medicine Finland, University of Helsinki, Helsinki, Finland; 6 Stanford University Center for Sleep Sciences, Palo Alto, CA, USA; 7 Wellcome Trust Sanger Institute, Hinxton, UK; 8 Analytic and Translational Genetics Unit, Department of Medicine, Massachusetts General Hospital, Boston, MA, USA; 9 Program in Medical and Population Genetics, Broad Institute of MIT and Harvard, Cambridge, MA, USA; 10 Psychiatric and Neurodevelopmental Genetics Unit, Department of Psychiatry, Massachusetts General Hospital, Boston, MA, USA; 11 Department of Clinical Neuroscience, Karolinska Institutet, Stockholm, Sweden

## Abstract

The pathophysiology of antisocial personality disorder (ASPD) remains unclear. Although the most consistent biological finding is reduced grey matter volume in the frontal cortex, about 50% of the total liability to developing ASPD has been attributed to genetic factors. The contributing genes remain largely unknown. Therefore, we sought to study the genetic background of ASPD. We conducted a genome-wide association study (GWAS) and a replication analysis of Finnish criminal offenders fulfilling DSM-IV criteria for ASPD (*N*=370, *N*=5850 for controls, GWAS; *N*=173, *N*=3766 for controls and replication sample). The GWAS resulted in suggestive associations of two clusters of single-nucleotide polymorphisms at 6p21.2 and at 6p21.32 at the human leukocyte antigen (HLA) region. Imputation of HLA alleles revealed an independent association with DRB1*01:01 (odds ratio (OR)=2.19 (1.53–3.14), *P*=1.9 × 10^-5^). Two polymorphisms at 6p21.2 LINC00951–LRFN2 gene region were replicated in a separate data set, and rs4714329 reached genome-wide significance (OR=1.59 (1.37–1.85), *P*=1.6 × 10^−9^) in the meta-analysis. The risk allele also associated with antisocial features in the general population conditioned for severe problems in childhood family (*β*=0.68, *P*=0.012). Functional analysis in brain tissue in open access GTEx and Braineac databases revealed eQTL associations of rs4714329 with LINC00951 and LRFN2 in cerebellum. In humans, LINC00951 and LRFN2 are both expressed in the brain, especially in the frontal cortex, which is intriguing considering the role of the frontal cortex in behavior and the neuroanatomical findings of reduced gray matter volume in ASPD. To our knowledge, this is the first study showing genome-wide significant and replicable findings on genetic variants associated with any personality disorder.

## Introduction

Antisocial personality disorder (ASPD) is a life-long condition involving habitual irresponsible and delinquent behavior, with prevalence of 1–3% in the general population, and 40–70% in prison populations.^1, 2, 3, 4, 5^ Previous twin and adoption studies report heritability estimates for ASPD up to 50%,^6, 7^ and several studies have attempted to unravel the genetic background of antisocial personality. Although men have consistently been found more often antisocial than women, it has been suggested that antisocial personality emerges from the same familial (including genetic and environmental factors) and non-familial influences in both sexes.^8^ Conduct disorder prior to age 15 is an essential diagnostic criterion for ASPD, and it markedly increases the risk for ASPD in adulthood.^9^ In a recent genome-wide association study (GWAS), Dick *et al.*^10^ found several markers with genome-wide significance associated with conduct disorder symptomatology, especially in the gene *C1QTNF7*, although none remained significant when individuals were classified dichotomously as cases and controls. Attention-deficit/hyperactivity disorder (ADHD) increases the risk for ASPD,^11^ and genes that have been previously found in association with ADHD have also been tested for association with ASPD. In a recent study, ADHD linked *SNAP25* polymorphisms (DdeI T/T, MnlI T/T haplotype) were associated with novelty-seeking scores in male ASPD subjects, and were more common in ASPD males when compared with sex-matched controls.^12^ Another candidate gene study revealed an association of *COL25A1* variant with comorbid ASPD and substance dependent in a subpopulation of African American and European American samples of substance-dependent patients with ASPD.^13^ In another recent study, a variant of a previously ADHD linked gene, *CDH13*, was associated with extreme violent behavior, and replicated in another Finnish cohort of violent offenders.^4, 14^ In the same study, the *MAOA* low-activity promoter genotype association with violent criminal behavior was replicated. MAOA deficiency was first reported in association with impulsive and aggressive behavior in a study of a Dutch family cohort,^15^ and later, *MAOA* low activity allele and childhood maltreatment interaction was linked to antisocial behavior.^16^ Recently, Tielbeek *et al.*^17^ reported the first genome-wide approach in the analysis of population-based samples of cases with antisocial behavior compared with population-based controls with only a little or no antisocial tendencies. Their study revealed no genome-wide significant associations, and the strongest association was observed in *DYRK1A* gene (*P*=8.70 × 10^−5^). In conclusion, although a number of studies have investigated antisocial behavior, no genome-wide significant or replicable findings on genes contributing to ASPD have been obtained thus far.

Here, our aim was to conduct the first GWAS in a cohort of prisoners fulfilling the diagnostic criteria of ASPD according to DSM-IV as compared to controls from the general population, and to replicate the most significant findings in another set of prisoners and controls. We then searched for the impact of the identified genetic risk for ASPD for antisocial features and its interaction with childhood risk environment in the general population. Finally, we investigated the functional implication of the risk variations for ASPD by searching for their correlations to gene expression in human tissue databases.

## Materials and methods

### Participants

**Figure 1:**
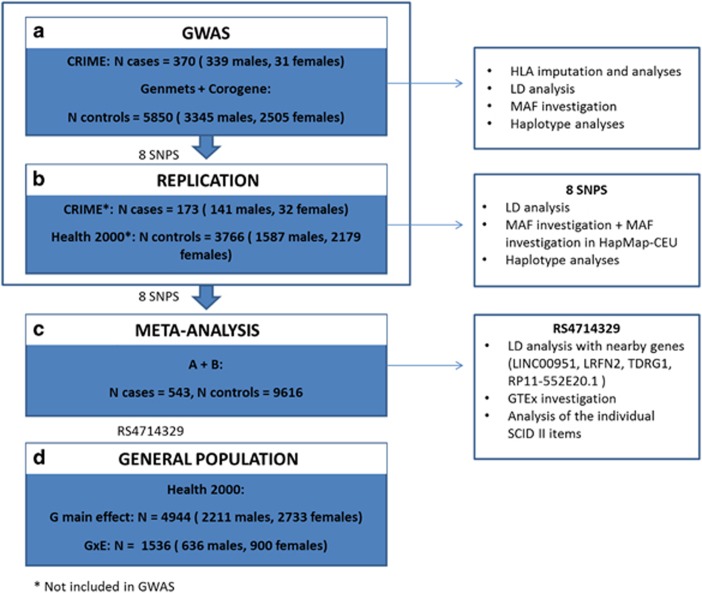
The flow-chart of the study. Box (**a**) illustrates the GWAS analyses including 370 cases (339 males, 31 females) and 5850 controls (3345 males, 2505 females) from the GenMets (sub sample of Health 2000 study) and Corogene (sub sample of FINRISK study) control samples. (**b**) On the basis of the GWAS results, eight variants were selected for analyses in the replication sample composed of those CRIME cases (*N*=173, 141 males, 32 females) and Health 2000 controls (*N*=3766, 1587 males, 2179 females) that were not included in the GWAS analyses. (**c**) The eight replication variants were included in the meta-analyses of the GWAS sample and the replication sample, including altogether 543 cases and 9616 controls. (**d**) The most significant hit of the analyses, rs4714329, was analyzed in the population sample for the association with antisocial features (*N*=4944, 2211 males, 2733 females), and for the gene x environment interaction of antisocial features and childhood adverse environment (*N*=1536, 636 males, 900 females). GWAS, genome-wide association studies. HLA, human leukocyte antigen; LD, linkage disequalibrium; MAF, minor allele frequency.

The study population including genotype data consisted of a total of 543 subjects with ASPD and 9616 participants from the general population. The clinical and sociodemographic characteristics of the participants in this study are shown in Table 1, and in the flow chart in Figure 1.

#### CRIME cohort

The Finnish CRIME sample was collected during 2010–2011, and it comprises 794 samples from all of the largest prisons in Finland. The sample collecting procedures have been described in more detail previously.^4^ In short, prisoners were screened for antisocial personality utilizing the Structured Clinical Interview Axis II (SCID-II) for the Diagnostic and Statistical Manual of Mental Disorders (DSM-IV). Sexual crime offenders, as well as individuals with diagnosis of psychosis, were excluded from the sample. Altogether, 568 (500 men, 68 women) criminal offenders fulfilled the criteria for antisocial personality disorder, whereas 196 were classified as non-antisocial, and 30 criminal offenders had an unknown ASPD status. Approximately 370 randomly selected individuals formed the discovery samples with GWAS data available, and 173 were included in the replication sample. The history of criminal convictions was obtained from the National Crime Register, and 77% of the antisocial prisoners were violent offenders having committed at least one violent crime. The remaining individuals of the ASPD sample were offenders who had been convicted for non-violent crimes, such as house break-ins or crimes against property. The history of substance abuse (alcohol, heroin, buprenorphine, amphetamine, cannabis and/or other) as well as childhood circumstances (for example, good circumstances, indifferent parents or severe maltreatment such as family violence) were screened with a questionnaire. Subjects provided a written informed consent. This study was approved by the Ethics Committee for Pediatrics, Adolescent Medicine and Psychiatry, Hospital District of Helsinki and Uusimaa, and Criminal Sanctions Agency. All the subjects who participated in the study received a voucher of 20 euros for their participation.

#### Control cohorts

The Health 2000 Study, including the GenMets sub cohort, and The National FINRISK Study, including the Corogene sub cohort, were used as control cohorts in the study (*N*=5850 with GWAS data available, used in the discovery phase; and *N*=3766 in the replication analysis; Supplementary Information).

### Phenotypes

#### Phenotype in GWAS, replication and secondary analyses of the ASPD sample

The subjects of the CRIME cohort, who fulfilled the criteria for ASPD according to DSM-IV and diagnosed by SCID-II were classified as cases. In addition, 22 individual items of the SCID-II questionnaire for ASPD were used in secondary analyses and analyzed individually. The phenotypes are described in detail in the Supplementary Information.

#### Phenotype in the secondary analyses of the population-based sample

The phenotype of antisocial features in the Health 2000 cohort was assessed with a scale, which is part of the home interview of the Health 2000 survey. The scale is originally from a 50-item scale, ‘Cook-Medley hostility scale',^18, 19^ and it included eight questions focusing on the aspects of deceitfulness, distrust to other people, and lack of empathy. Information on the childhood environment of economic difficulties or severe conflicts in the family were questioned and applied in gene-environment interaction analyses (Supplementary Information).

### Genotyping and statistical analyses

#### Genotyping

Illumina Human670QuadCustom Beadchip and HumanHap610-Quad SNP array (Illumina, San Diego, CA, USA) were used for genotyping of common single-nucleotide polymorphisms (SNPs) genome-wide at the Welcome Trust Sanger Institute, Cambridge, UK for the CRIME and control cohorts, respectively. Stringent quality control was used and only SNPs originally included in all data sets of controls and cases were analyzed, resulting in 481866 SNPs in the final data set. Targeted genotyping for both controls and cases were performed by Sequenom MassARRAY iPLEX technology (San Diego, CA, USA) simultaneously at the Institute for Molecular Medicine, Finland (for details on genotyping, see Supplementary Information).

#### HLA-imputation

Imputation of the GWAS genotype data with the classical human leukocyte antigen (HLA) alleles was performed utilizing the HIBAG software^20^ (for more information, see Supplementary Information).

#### Statistical analyses and linkage disequilibrium analyses

The power calculations were performed utilizing Genetic Power Calculator (http://pngu.mgh.harvard.edu/~purcell/gpc/).^21^ All of the association analyses were performed using a generalized linear (logistic) model with PLINK v1.07 (http://pngu.mgh.harvard.edu/~purcell/plink/)^22^ with age and sex and the 10 first multidimensional scaling clusters as covariates. The meta-analyses with fixed effect model were performed with GWAMA software (http://www.geenivaramu.ee/en/tools/gwama.^23^ SPSS (IBM Released 2013. IBM SPSS Statistics for Windows, Version 22.0. Armonk, NY, USA). The linkage disequilibrium (LD) and haplotype analyses were performed utilizing PLINK v1.07, Haploview,^24^ and SNAP (https://www.broadinstitute.org/mpg/snap/).^25^ Detailed description of the statistical analyses can be found in the Supplementary Information.

### Gene expression analyses

The GTEx Portal (http://www.gtexportal.org/home/)^26^ and The Braineac—the Brain eQTL Almanac (http://www.braineac.org/) were utilized to investigate correlations between variant genotype and brain- and testis-tissue gene-expression levels. More information can be found in the Supplementary Information.

## Results

### GWAS for ASPD

**Figure 2:**
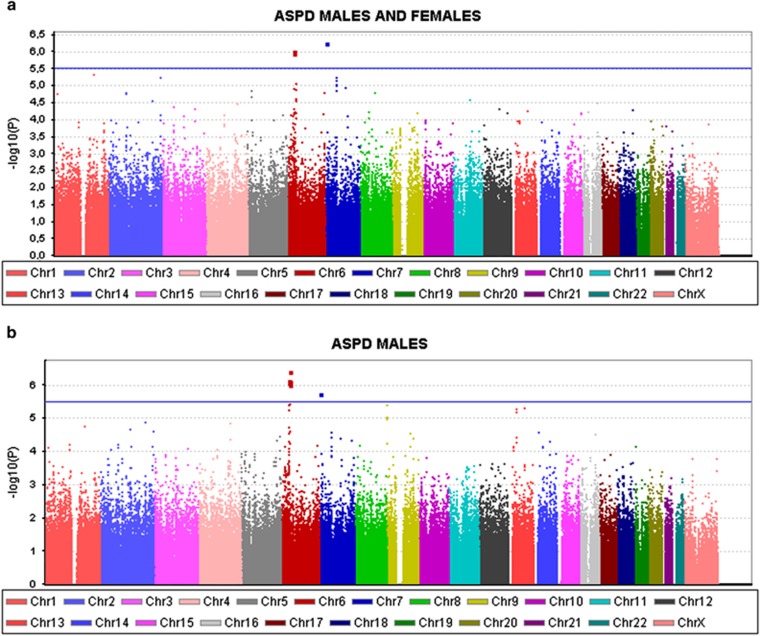
Manhattan plot of the entire sample of males and females combined in the analysis of the ASPD cases versus population-based controls (**a**) and for the males' sub-sample (**b**). ASPD, antisocial personality disorder.

We conducted genome-wide association analyses, for DSM-IV based ASPD, using the entire GWAS study sample (*N* cases=370, *N* controls=5850), as well as in the sample including only males (*N* cases=339, *N* controls=3345). The number of the female cases was too small (*N*=31) to perform a separate analysis. The Manhattan plots for the genome-wide analyses are presented in Figure 2, illustrating the results of the analyses for the whole sample, and the sample including only males, respectively. Supplementary Tables 1a and 1b display the most significant 50 variants associated with antisocial personality disorder in the combined sample, and in males, respectively. None of the associations achieved genome-wide significance (*P*<5.0 × 10^−8^). However, in the quantile-quantile (Q–Q) plots for the observed versus expected results (−10 log(*P*-value)), for both analyses, several data points were observed above the lift of line indicating more significant associations than expected by chance (Supplementary Figures 1a and b).

The strongest signal for association in the combined analysis of males and females was detected in chromosome 7p22.2, in the vicinity the *SDK1* gene, for rs6462756 (odds ratio (OR)=1.84 (1.45–2.33), *P*=5.5 × 10^−7^). As no other variant within 500 kb (250 kb down/upstream, including 135 SNPs) of it gave any signal, it was considered spurious, and was not selected for replication in this study. The next strongest associations were observed for rs9268528 (OR=0.58 (0.46–0.72), *P*=9.9 × 10^−7^) and rs9268542 (OR=0.58 (0.46–0.72), *P*=1.1 × 10^−6^), located on chromosome 6p21.32, intragenic of the *BNTL2* and *HLA-DRA* genes. These two variants were selected for replication, together with an additional nearby intragenic variant, rs2395163 (OR=0.59 (0.46–0.77), *P*=6.2 × 10^−5^), and an intronic variant of *HLA-DRA* gene, rs2239804 (OR=0.61 (0.49–0.77), *P*=1.2 × 10^−5^). All the association signals of these variants in the vicinity of the *HLA-DRA* gene originated from the major allele. Supplementary Figure 2a shows the regional Manhattan plot of chromosome 6 for the analysis of the whole sample.

In the analysis of the males alone, the strongest association signals were observed for variant rs6458146 (OR=1.72 (1.40–2.13), *P*=3.9 × 10^−7^), residing in an intergenic region on chromosome 6p21.2. An additional four markers in nearby loci gave signal with *P*-values<5 × 10^−5^ (rs9471290, OR=1.68 (1.37–2.06), *P*=8.1 × 10^−7^; rs10498746, OR=1.72 (1.38–2.13), *P*=9.9 × 10^−7^, rs7749170, OR=1.67 (1.35–2.08), *P*=3.8 × 10^−6^; rs4714329, OR=1.56 (1.27–1.92), *P*=2.5 × 10^−5^). Supplementary Figure 2b shows the regional plot for the males' association analysis.

Overall, our findings suggest altogether eight variants that associated with ASPD, originating from distinctive regions located at 6p21 (6p21.2 and 6p21.32). These were chosen for the regenotyping and for the replication analyses.

### Regenotyping, replication and meta-analysis

#### Re-genotyping and confirmatory analyses

In order to confirm the consistency of the genotypes from the GWA analyses, we performed simultaneous regenotyping of the selected eight variants both in the CRIME sample as well as in the GenMets controls from Health2000 (Genmets sample) by another technology (Sequenom MassARRAY iPLEX). There was a >99% similarity between the original genotypes from GWAS in the discovery analysis and in the regenotyping (Supplementary Information). Thus, the original signals from the GWAS on 6p21.2 and 6p21.32 were not due to spurious genotyping errors.

We also compared the minor allele frequencies (MAFs) of these variants in our cases, as well as their controls from the HapMap-CEU population (http://www.ncbi.nlm.nih.gov/SNP/) (Supplementary Table 4). The MAFs for all these eight SNPs were relatively consistent between the population controls of GWAS and of replication samples. When looking the genetic variation at these chromosomal regions more widely, the MAFs for the variants on 6p21.2 were well in line with the HapMap-CEU. The MAFs of the variants near the *HLA-DRA* gene, on 6p21.32, were considerably lower in both of the GWAS and replication population controls as compared with the HapMap-CEU. This is likely to reflect the genetically complicated nature of that particular chromosomal region with widely extending structures of LD and with high variation in different populations.

#### Replication analysis

Table 2 shows the results among the discovery sub-cohort (*N* cases=370, *N* controls=5850) and the replication sub-cohort (*N* cases=173, *N* controls=3766), and the entire study population (meta-analysis; *N* cases=543, *N* controls=9616). The variants on 6p21.2 showed a consistent signal in the replication analysis (rs7749170 failed in the Sequenom genotyping and is therefore absent), whereas the results for the SNPs on 6p21.32 were diluted or reversed when compared to the GWAS signal. Two out of four of the SNPs on 6p21.2 showed a statistically significant signal in the replication analysis (males and females combined). The most significant signals on 6p21.2 were revealed for rs4714329 (OR=1.75 (1.37–2.24), *P*=9.0 × 10^−6^) and for rs9471290 (OR=1.40 (1.09–1.81), *P*=8.3 × 10^−3^). The replicating variants were included in a meta-analysis in which rs4714329 yielded an OR of 1.59 (1.37–1.85) and *P*-value of 1.6 × 10^−9^, and rs9471290 had an OR of 1.49 (1.28–1.73) and a *P*-value of 2.9 × 10^−7^. A *post hoc* replication analysis of rs4714329 in the female sample (*N* cases=32, *N* controls=2179) revealed an OR of 2.55 (1.50–4.33) and a *P*-value of 5.3 × 10^−4^. Thus, we detected a replication with two variants at 6p21.2, the most robust finding with rs4714329 reaching genome-wide significance (*P*=1.6 × 10^−9^ in the meta-analysis), although there was evidence that variants on 6p21.32 were weaker according to the replication study.

### LD and haplotype analyses on 6p21.2 and 6p21.32

#### LD and haplotype analysis on 6p21.2

To investigate whether the most significant variant from the meta-analysis (rs4714329) belonged to a longer haplotype, we performed LD and haplotype analyses for all of the four SNPs residing in the 6p21.2 genomic region. Two of the variants (rs6458146 and rs10498746) were located in the same haploblock (bp 40218128–40224268, GRCh37; Supplementary Figure 3). Also, rs9471290 (bp 40260515) was in a strong LD with those (D′=0.93 and 0.95, and LOD⩾2.0, respectively). Rs4714329 was, however, only in a moderate LD with the other 6p21.2 variants (D′=0.77, 0.68, and 0.66 to rs9471290, rs6458146 and rs10498746, respectively). A *post hoc* haplotype analysis of the SNPs with the most significant signal from the replication analysis yielded an association signal from a relatively frequent (*f*=0.31) haplotype of minor alleles A–G (rs9471290 and rs4714329, respectively) of OR=1.59 (*P*=5.6 × 10^−6^) in the GWAS data, and of OR=1.49 (*P*=0.0022) in the replication cohort (males and females combined). Thus, the signal for association from 6p21.2 was not strengthened by the haplotype and the risk genotype was best captured by the G-allele of rs4714329.

In an effort to further elucidate the possible origin of the signal of our best hit, we investigated the LD between rs4714329 and the variants within the nearby genes (*RP11-552E20.1*, *TDRG1*, *LINC00951* and *LRFN2*) utilizing the GWAS variant data of the CRIME cohort as well as the HapMap3 project data and the 1000 Genomes Pilot 1 data (Supplementary Information). The strongest LD was observed between rs4714329 and several variants of the *LINC00951* gene, consistently in all three data sets. In addition, the CRIME sample *LINC00951* gene variants (rs17619142 and rs17619309) revealed nominal association with ASPD (OR of 1.18 and 1.5, and *P* value of 0.002 and 0.159, respectively), which supported the role of *LINC00951* as the strongest candidate of the nearby genes for the signal origin.

#### LD analysis on 6p21.2 and 6p21.32

As stated earlier, the HLA region at 6p21.32 is known for its complexity and extended LD ranging across multiple HLA and non-HLA genes in the region, and the HLA alleles may be captured by tag SNPs even outside the region.^23^ As the two most significant sets of variants with leading SNPs (rs4714329 at 6p21.2 and rs9268528 at 6p21.32) resided only approximately 8 Mb distance from each other, we investigated whether there would be LD between them. We also analyzed their haploblock structures, and which other SNPs would tag them in the data. Although the variants were strongly linked within the set, especially on the 6p21.32 loci, there was no LD between the two sets. Furthermore, they did not share same haploblocks, and no tagging was evident across the two sets (data not shown).

#### Analysis of HLA alleles at 6p21.32

**Figure 3:**
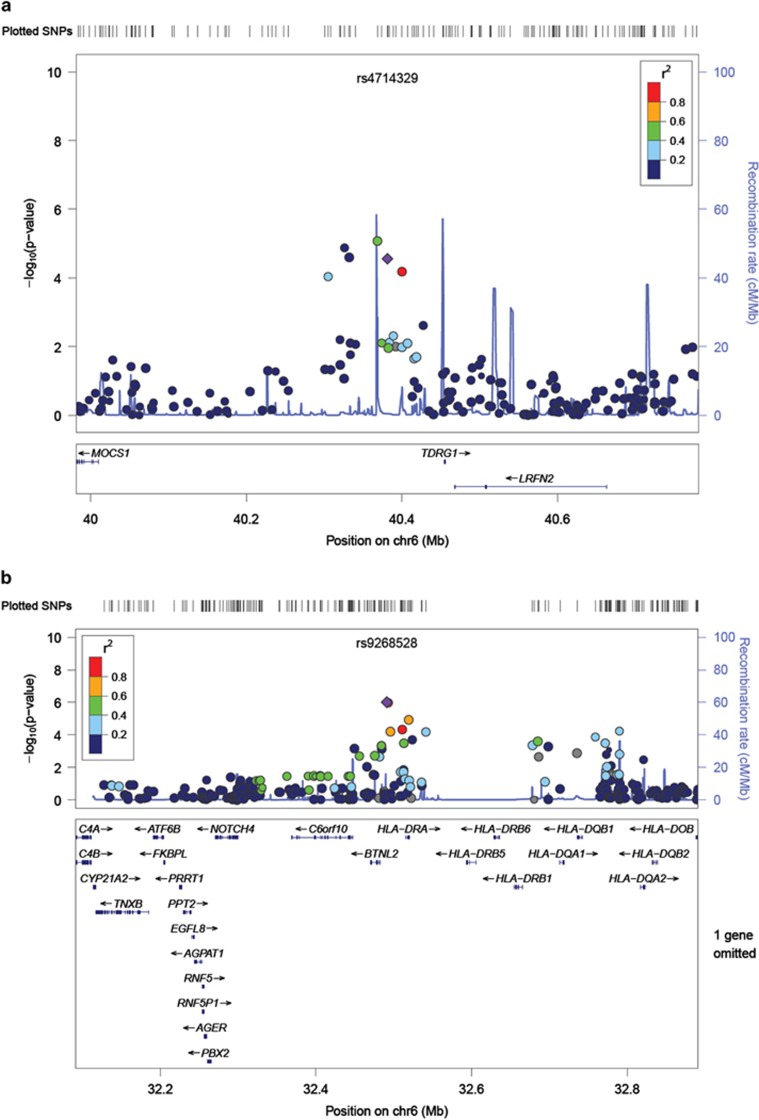
Association peaks at the chr 6 LINC00951 (**a**) and HLA (**b**) regions. Extended LD over the HLA class II region can be observed in **b**. HLA, human leukocyte antigen; LD, linkage disequalibrium; SNP, single nucleotide polymorphism.

The HLA region is highly polymorphic and individual SNPs at the HLA region are commonly part of more than one HLA allele and haplotype. The GWAS analysis of the associating locus (6p21.32) at the HLA region showed multiple SNPs associating with ASPD and the LD was carried over from *BTNL2* gene to as far as the *HLA-DQB1* gene region (Figure 3b). We observed that there were no directly genotyped SNPs at the *HLA-DRB1* locus that would conceivably have depicted whether the highest association was indeed over *HLA-DRA* or perhaps over the *HLA-DRB1* locus. It was also possible that some of the associating SNPs tagged an independent HLA allele, or that specific HLA alleles or their coding polymorphisms, rather than single SNPs, would drive the association with ASPD. The HLA region is also known to be difficult for probe designing or to impute with SNPs, therefore, we imputed the GWAS genotype data with the classical HLA alleles (Supplementary Information), and studied whether specific HLA alleles would associate with ASPD. We detected four HLA alleles at *HLA-DRB1* and *HLA-DQA1* genes with similar significance as individual SNPs but with higher OR in association with ASPD (Supplementary Table 2). The strongest associations were seen with a common *HLA-DRB1* allele DRB1*01:01 (OR=2.19 (1.53–3.14), *P*=1.9 × 10^−5^, *f*=0.17) and with DQA1*01:01 (OR=2.09 (1.46–2.99), *P*=5.6 × 10^−5^, *f*=0.17) that are known to be in tight LD (current data *r*^2^=0.981). In addition, protective associations with DRB1*04:04 and DQB1*03:02 alleles were detected with similar protective effects as the strongest individual SNPs of the GWAS analysis (DRB1*04:04 OR=0.39 (0.18–0.57), *P*=9.7 × 10^−5^, *f*=0.04 and DQB1*03:02 OR=0.52 (0.37–0.74), *P*=2.5 × 10^−4^, *f*=0.11). To investigate whether the HLA allele effects were directly explained by the individual SNPs, we conditioned the analysis for the strongest variant at the HLA region (rs9268528), which failed to remove the association with DRB1*01:01 (conditioned *P*=2.3 × 10^−3^). Similarly, conditioning for the strongest four HLA alleles failed to remove the association with rs9268528, suggesting independent roles for the HLA alleles and rs9268528 in ASPD.

As individual amino acids coded by the HLA genes have been previously indicated to drive disease association,^27, 28^ we examined whether the associations of the top SNPs could be explained by individual coding polymorphisms at the *HLA-DRB1* or *DQA1* alleles (Supplementary Table 3). The DRB1 position 11 V was observed with the strongest protection for ASPD (OR=0.49 (0.37–0.66), *P*=2.4 × 10^−6^). Interestingly, DRB1 position 11 V is detected here with those DRB1*04 alleles that were associated at the allele level. In addition, DRB1 position 11 V was in relatively high LD with rs2395163 (D′=0.83, *r*^2^=0.56). Conditioning for position 11 V removed the association with rs2395163, but not with the leading variant at the HLA region (rs9268528 conditioned *P*=8.3 × 10^−3^) or with DRB1*01:01 (conditioned *P*=5.2 × 10^−4^), which was the strongest association at the HLA-region after conditioning for DRB1 position 11 V. Finally, we studied whether DRB1 position 11 V, together with HLA-DRB1*01:01, would explain the association with rs9268528. This was not the case. Even though the *P*-value for rs9268528 association was weaker after conditioning for both HLA-DRB1*01:01 and for DRB1 position 11 V, it was still significant with a point-wise *P*-value<0.05.

### Analyses for sensitivity and the individual SCID-II items

#### Association sensitivity

Given that the main focus of this study is ASPD, cases were selected from the whole of the CRIME cohort based on the SCID-II diagnostics. However, the non-ASPD criminals group may include cases near the ASPD diagnosis cut-off threshold. Thus, we performed an analysis on non-ASPD status (all males, *N*=129) to examine whether the signal from the selected eight variants was specific for ASPD or if it covered a broader range of criminal activity. No association signal was detected for any of the selected variants. As substance abuse, as well as violent behavior, is linked to antisocial personality disorder, we performed sensitivity analyses including only those ASPD cases with substance abuse (*N*=358, males=327) or violent crime (*N*=285, males=265). Among substance abusers, a slight strengthening of the signal for the variants near the *HLA-DRA* gene on 6p21.32 was observed, but the signal was diluted for the variants on 6p21.2 (data not shown). Among violent criminals, the signals from all of the variants were diluted (data not shown).

#### Individual SCID-II items

ASPD diagnosis is based on a relatively broad range of antisocial behavioral characteristics. To elucidate the origin of the strong association signal arising from rs4714329 to ASPD, we analyzed each 22 characterizing items of the SCID II questionnaire individually (Supplementary Information; page 1, Supplementary Tables 7 and 8 and Supplementary Figure 5). The odds ratios for all of the 22 questions were quite similar, ranging between 1.4 and 1.7. The most significant association was achieved for item (20), ‘Reckless disregard for safety of self or others' with the OR of 1.66 (1.41–1.94) and a *P*-value of 6.0 × 10^−10^ (heterogeneity index *I*^2^=0.44 (scale 0–1), indicating more variation than expected by chance) in the meta-analysis (*N* cases=477, *N* controls=9616). The most significant association with the heterogeneity index value of zero was observed for item (16), ‘Failure to conform to social norms with respect to lawful behaviors, as indicated by repeatedly performing acts that are ground for arrest' (OR=1.60 (1.37–1.87), *P*=1.7 × 10^−9^, *N* cases=529, *N* controls=9616). The most significant items typically included >400 individuals each, whereas some of the items, such as items (5) or (8) only included <100 individuals, hence, the *P*-values here should preferably be interpreted as a reflection of power. Consequently, rs4714329 was found to be broadly associated with the different aspects of ASPD.

### Association of rs4714329 to antisocial features in general population

The prevalence of ASPD is low in the population (1–3%),^1^ with a well-established role of early adverse environment in its etiology.^29, 30^ We hypothesized that the most significant hit in the ASPD analyses, the risk allele G of rs4714329, would also have an impact on antisocial features in the general population, especially among those individuals who had encountered severe difficulties in their childhood. Thus, we tested the risk variant in the Health 2000 population sample for association with antisocial tendencies, and also performed a gene × environment interaction analysis, where reported severe conflicts or economic difficulties in the childhood family were considered as a risk environment. As a measure of antisocial features, we utilized a scale assessed in the Health 2000 survey and focusing on deceitfulness, distrust to other people, and lack of empathy (see Supplementary Information for phenotype in the secondary analysis of the population-based sample). No signal was detected for the main effect of antisocial features in the complete sample (males and females: *N*=4944, *β*=0.13, *P*=0.175; males only: *N*=2211, *β*=0.203, *P*=0.143). However, there was a modest signal for interaction between the risk environment and rs4714329 in males, but not in females, in the general population (males: a *β* of 0.647, and a *P*-value of 0.045 for the interaction term), and the risk allele G associated significantly with antisocial features among males with the childhood risk environment (*N*=636, *β*=0.68, *P*=0.012).

### Expression analysis of rs4714329 in brain tissue databases

The most significant variant of the replication and meta-analyses for ASPD, rs4714329 resides in the proximity of the genes *LINC00951*, *LRFN2*, *RP11-552E20.1* and *TDRG1*. *LINC00951* and *LRFN2* are expressed mostly in the brain, especially in the cortex and testis. *TDRG1* gene is expressed mostly in testis and in the brain. Thus, we investigated in the GTEx Portal and in the Braineac database the possible association of rs4714329 with the nearby residing genes' expression levels in all of the available brain tissues and in testis (testis only in the GTEx Portal; Supplementary Information).

In the GTEx Portal, the most significant association was achieved with *LINC00951* gene expression in cerebellum (*N*=103) with the *β* of 0.51 and a *P*-value of 2.0 × 10^−6^. *LRFN2* and *TDRG1* gene expressions revealed association in the cerebellar hemisphere (*N*=89; *P*-value=2.0 × 10^−5^ and *β*=0.56, *P*=4.0 × 10^−5^ and *β*=0.51, respectively; Supplementary Table 6a). In Braineac, no data were available for *LINC00951,* but rs4714329 was associated most significantly (*P*=7.4 × 10^−4^) with *LRFN2* expression in cerebellum (*N*=130), although consistently with the GTEx Portal, *LRFN2* is mostly expressed in the cortex. For *TDRG1* expression in Braineac, the most significant (*P*=3.8 × 10^−3^) association was observed in occipital cortex (OCTX, *N*=129; Supplementary Table 6b). Neither databases had data available for *RP11-552E20.1* gene expression; in Braineac, no data were available for *LINC00951*, and *RP11-552E20.1* genes. Thus, the results revealed that rs4714329 associated to expression of the nearby located genes in brain so that the risk allele G of rs4714329 was found to correlate with reduced levels of *LINC00951, LFRN2* and *TRG1* mRNAs in cerebellum and of *TDRG1* in occipital cortex.

## Discussion

The results from this first GWAS on ASPD reveal genome-wide significant and replicable associations for variants residing on chromosome 6p21.2. According to our linkage investigation, our best hit, rs4714329 is in considerable LD with several polymorphisms of the *LINC00951* gene, and not up to the same level with the polymorphisms of the other genes in that genomic region, suggesting that the *LINC00951* gene is the strongest candidate for the signal origin. However, analysis of the gene expression data from the GTEx and Braineac databases revealed equally strong associations between rs4714329 and the expression levels of three nearby genes, namely *LINC00951*, *LRFN2* and *TDRG1*, in tissues from cerebellum, of which the *LINC00951* association was the strongest in the cerebellum and *LRFN2* in the cerebellar hemisphere. Although cerebellum has traditionally been linked to motor control, it has also been suggested to be involved in cognition^31^ and personality,^32^ as well as autism spectrum disorders^33^ and psychosis.^34^ Most interestingly, Leutgeb *et al.* reported an altered cerebellum–amygdala connectivity among violent offenders, although their study excluded psychopathic or personality-disordered individuals.^35^ Although there was no correlation of rs4714329 to RNA levels in frontal cortex, it is highly interesting that the general levels of the transcripts for both *LINC00951* and *LRFN2* are particularly high in frontal cortex,^36, 37^ as the reduced GMV at that particular cortical region is one of the most consistently-reported biological findings in ASPD.^38, 39^

Chromosomes 6p21.2 and 6p21.32 belong to the chromosomal region of major histocompatibility complex, and have been previously linked to psychiatric disorders including schizophrenia^6, 40^ and bipolar affective disorder,^41^ as well as neurologic disease such as photosensitive epilepsy,^42^ late-onset Alzheimer disease^43^ and restless legs syndrome.^44^ In this study, we discovered novel suggestive associations of HLA-DRB1*01:01 and DRB1*04:04 with ASPD. Previous studies have reported the same alleles in association with schizophrenia,^45, 46, 47^ whereas other studies have not been able to replicate those findings^48^ and the link has remained controversial. In this study, additional analysis of coding polymorphisms suggested an independent role for Valine at position 11 on DRB1 that was tagged by one of the most significant GWAS hits in this study, rs2395163. The findings suggest that ASPD may share genetic risk factors in this locus with other psychiatric and neurological disorders.

The study sample in this study overlapped partially with the sample in a previous study of extreme violent behavior.^4^ However, the significant variants were not associated with violent offending in general, which indicates that the variants were associated with ASPD *per se*. This conclusion is also supported by the finding on antisocial attitudes observed in the cohort from the general population, where the risk allele was found to increase the presence of the antisocial features of deceitfulness and lack of empathy among those individuals who reported severe family conflicts or economic difficulties in their childhood families.

The ASPD diagnosis is controversial, since many clinicians and researchers think that the category is too heterogeneous and has overlap with other disorders.^30^ ASPD and other personality disorders have been reported to have comorbidity with psychopathy, a personality trait tightly connected to criminality and forensic psychiatry.^5, 49^ Although ASPD and psychopathy diagnoses are based on different assessment tools, the distinction between those two is not completely clear.^50, 51^ We consider our study sample relatively homogenous as the participants were all criminal offenders and had a convergent diagnosed ASPD. However, to further study the behavioral origin of our genome-wide significant hit, rs4714329, we investigated its association individually with each of the SCID II items used in this study. All of the 22 items gave consistent ORs, indicating that the original signal covers all the different aspects of ASPD according to DSM-IV, supporting the homogeneity of the study sample. However, we would like to point out, that the most significant associations were observed for those items that particularly describe social learning. Association with weak learning from social feedback would seem very logical for different frontal cortex genetic regulation, but it may also reflect those in cerebellum. It has also been reported that 21–45% of the prison inmates have a comorbid ADHD,^52, 53^ and a shared etiology has been suggested for such externalizing phenotypes as adult substance use and antisocial personality, and childhood conduct disorder and ADHD.^54, 55, 56^ ADHD was not diagnosed in our study sample, however, ~96% of the analyzed sample were substance abusers. The externalizing dimension of behavior is hypothesized to reflect an inherited predisposition for developing one or more of the aforementioned disorders.^57^ Thus, the previous findings on reduced GMV in frontal brain areas associated with ASPD may also reflect the comorbidity of the externalizing problems, consistent with the findings of ADHD prevalence in prisoners and supported by our findings here.

There were limitations in the study. In the GWAS study, the CRIME sample was genotyped with a different array than the control samples (GenMets, Corogene), although in the same facility of Welcome Trust Sanger Institute. Therefore, only the SNPs that overlapped in all of the three genotyping data sets were included in the analyses and even unusually stringent thresholds were used in the quality control. Furthermore, the regenotyping of the eight replication variants in the CRIME subjects and GenMets controls that were included in the GWAS, revealed practically identical genotypes as the GWAS genotyping with >99% concordance between the two methods. In a *post hoc* repeat GWAS analysis, including the CRIME subjects and GenMets controls, the results were consistent with the original analysis. This also indicated that had there been discrepancy with the Corogene control sample genotypes of the eight SNPs in the GWAS, this would have shown in the repeat analysis results. In spite of intensive QC, spurious associations may occur. We considered the top variant (rs6462756, near *SDK1* gene) of the analysis of males and females combined as a plausible false positive, as it was the only signal from that genomic region (7p22.2), where the SNP density was not particularly sparse, rather to the contrary, relatively dense (35 SNPs per 100 kb, compared with chr 7p average 21 SNPs per 100 kb and the nearby 500 kb average 25 SNPs per 100 kb), and thus, it was not selected to the regenotyping nor to the replication study.

Another factor raising concerns regards the reversed replication results of the set of hits on 6p21.32, near *HLA-DRA* gene. A possible explanation may lie in the high gene density and polymorphism rate of the HLA region, combined with the lack of power in this study. The MAF investigation of the eight variants, including the HapMap-CEU population sample, revealed considerable MAF variance for the *HLA-DRA* variants, but not for the variants on 6p21.2, near to the *LINC00951* gene. The only variant in the HLA-DR region on 6p21.32 showing similar MAF in discovery and replication was rs2395163 that tagged a relatively rare HLA-DRB1*04:04 association. As the allele frequency of HLA-DRB1*04:04 was only 0.04, it was not surprising that the association was not replicated in the relatively small replication sample. However, it is important to emphasize that the *HLA*-findings emerge only from the imputed GWAS data, and no replication was addressed in this study.

These findings are limited to the Finnish population-based cohorts, and therefore replications in other samples of other populations should be pursued. With altogether 543 cases and 9616 controls, our study remains very much underpowered, as indicated in the power calculations. Bigger sample sizes are needed, although collection of large samples is rather demanding, and especially for behavioral phenotypes of a relatively special nature, such as ASPD, large samples may materialize at the expense of phenotype homogeneity. The large GWAS's have proven the method a useful tool for identifying genetic loci in complex disease of small individual factor effect sizes, such as in schizophrenia.^34^ However, GWAS in smaller samples may also reveal important associations, particularly, when the phenotype is accurate, and the suggestive findings may be of value for future studies with larger sample sizes.

The results of this study need to be interpreted carefully. There are examples of how the genetic associations of ASPD may be or have been misused for prediction purposes or in courthouses.^58, 59^ The concept of heritability is sometimes misinterpreted. When ASPD is described as 50% heritable, it implicates that heritability explains 50% of the variance in that population in which the study was conducted in, and not that on the individual level an ASPD subject had inherited 50% of the disorder from their parents and 50% was due to the environmental influences. The findings of this study cannot be implemented for any prediction purposes, or brought into courthouses to be given any legal weight. Instead, this study serves well as a candidate variant reservoir for personality traits and disorders adding to the few candidate variants that have previously existed for ASPD. Important next steps would be to discover the causal factors tagged by the findings here and to elucidate the molecular functions involved with ASPD. In conclusion, our results showed a robust signal for several variants located beside *LINC00951* with potential functional effects. To our knowledge, this is the first study showing genome-wide significant and replicable findings on genetic variants associated with any personality disorder.

## Figures and Tables

**Table 1:** The clinical and sociodemographic characteristics of the participants

	*ASPD (violent criminals, substance abuse, maltreatment)*	*Controls*
*GWAS (*N*)*		
Males	339 (265 (NA=4), 327, 89 (NA=4))	3345
Females	31 (20, 31, 13 (NA=2))	2505
All	370 (285 (NA=4), 358, 102 (NA=6))	5850
Age (mean±s.d.)	34.5±8.0	57.4±13.2
		
*Replication cohort (*N*)*		
Males	141 (107 (NA=2), 134, 37 (NA=2))	1587
Females	32 (15 (NA=2), 27, 7	2179
All	173 (122 (NA=4), 161, 44 (NA=2))	3766
Age (mean±s.d.)	34.2±9.2	55.0±17.0

Abbreviations: ASPD, antisocial personality disorder; GWAS, genome-wide association study; NA, data not available.

In the GWAS cohort, data were unavailable for the ASPD cases for four individuals (all males) on their violent crimes, and for six individuals (four males, two females) on their childhood maltreatment. In the replication cohort, the number of individuals lacking the information on violent crimes was four (two males, two females), and on their childhood maltreatment two (both males).

**Table 2:** The results for the eight best hits in Discovery Cohort (GWAS) and Replication Cohort (Sequenom Genotyping)

*CHR*	*SNP*	*BP*	*Gene (nearest gene)*	*A1*	*A2*	*Discovery (GWAS, CRIME vs GENMETS and COROGENE)*						*Replication (CRIME vs HEALTH-2000, excluding GENMETS)*						*Meta-analysis (discovery and replication)*			
						N *cases=370,* N *controls=5850*						N *cases=173,* N *controls=3766*									
						*OR*	*L95*	*U95*	P-*value*	*MAF cases*	*MAF controls*	*OR*	*L95*	*U95*	P-*value*	*MAF cases*	*MAF controls*	*OR*	*L95*	*U95*	P-*value*
*ASPD males and females*																					
6	rs4714329	40273457	*LINC00951*	G	A	1.5	1.24	1.81	2.78E−05	0.443	0.3855	1.75	1.37	2.24	9E-06	0.5087	0.3777	1.59	1.37	1.85	1.64E−09
6	rs9471290	40260515	*LINC00951*	A	G	1.53	1.27	1.85	8.51E−06	0.43	0.3667	1.4	1.09	1.81	0.00828	0.4393	0.3571	1.49	1.28	1.73	2.88E−07
6	rs6458146	40218128	*LINC00951*	G	A	1.53	1.26	1.85	1.32E−05	0.393	0.327	1.21	0.93	1.57	0.1504	0.3555	0.3172	1.41	1.21	1.64	1.28E−05
6	rs10498746	40224268	*LINC00951*	G	A	1.53	1.25	1.86	2.46E−05	0.353	0.2809	1.14	0.88	1.49	0.3239	0.3035	0.2704	1.38	1.18	1.61	7.12E−05
6	rs2395163	32387809	*HLA-DRA*	G	A	0.59	0.46	0.77	6.23E−05	0.137	0.1903	0.83	0.61	1.14	0.2438	0.1532	0.1883	0.68	0.56	0.83	1.26E−04
6	rs2239804	32411523	*HLA-DRA*	G	A	0.61	0.49	0.76	1.19E−05	0.25	0.3219	1.3	1.02	1.65	0.0341	0.3815	0.3147	0.86	0.73	1.01	0.06902
6	rs9268528	32383108	*HLA-DRA*	G	A	0.58	0.46	0.72	9.89E−07	0.249	0.3296	1.38	1.09	1.75	0.00794	0.4017	0.3209	0.86	0.74	1.02	0.07626
6	rs9268542	32384721	*HLA-DRA*	G	A	0.58	0.46	0.72	1.12E−06	0.25	0.3307	1.42	1.12	1.8	0.00373	0.4104	0.3228	0.88	0.75	1.03	0.1139
																					
*ASPD males*																					
						*N* cases=339, *N* controls=3345						*N* cases=141, *N* controls=1587									
6	rs9471290	40260515	*LINC00951*	A	G	1.68	1.37	2.06	8.05E−07	0.438	0.366	1.37	1.03	1.83	0.03029	0.4397	0.3573	1.57	1.33	1.85	1.33E−07
6	rs4714329	40273457	*LINC00951*	G	A	1.56	1.27	1.92	2.52E−05	0.445	0.3833	1.57	1.19	2.08	0.00151	0.4894	0.374	1.56	1.32	1.85	1.38E−07
6	rs6458146	40218128	*LINC00951*	G	A	1.72	1.4	2.13	3.86E−07	0.404	0.3217	1.22	0.91	1.63	0.1934	0.3582	0.3144	1.53	1.29	1.82	1.01E−06
6	rs10498746	40224268	*LINC00951*	G	A	1.72	1.38	2.13	9.94E−07	0.364	0.276	1.18	0.88	1.59	0.2736	0.3121	0.2744	1.51	1.27	1.8	4.04E−06
6	rs2395163	32387809	*HLA-DRA*	G	A	0.52	0.39	0.69	5.51E−06	0.128	0.1931	0.8	0.56	1.14	0.2181	0.1489	0.1875	0.61	0.49	0.77	1.59E−05
6	rs2239804	32411523	*HLA-DRA*	G	A	0.57	0.45	0.72	3.96E−06	0.245	0.3283	1.22	0.93	1.61	0.1459	0.3723	0.3141	0.79	0.66	0.95	0.01264
6	rs9268528	32383108	*HLA-DRA*	G	A	0.54	0.43	0.69	7.49E−07	0.246	0.3377	1.3	0.99	1.7	0.05452	0.3936	0.3198	0.8	0.67	0.96	0.01649
6	rs9268542	32384721	*HLA-DRA*	G	A	0.55	0.43	0.69	8.52E−07	0.248	0.3392	1.3	1	1.7	0.0519	0.3972	0.322	0.81	0.67	0.96	0.01874
																					
*ASPD females*																					
						*N* cases=31, *N* controls=2505						*N* cases=32, *N* controls=2179									
6	rs4714329	40273457	*LINC00951*	G	A	1.21	0.69	2.12	0.507	0.419	0.3884	2.55	1.5	4.33	0.00053	0.5938	0.3804	1.79	1.22	2.63	0.00292
6	rs9268542	32384721	*HLA-DRA*	G	A	0.65	0.35	1.21	0.1727	0.274	0.3194	1.94	1.17	3.23	0.01049	0.4688	0.3233	1.24	0.84	1.84	0.27272
6	rs10498746	40224268	*LINC00951*	G	A	0.69	0.37	1.29	0.2462	0.226	0.2874	1	0.56	1.79	0.9975	0.2656	0.2676	0.84	0.55	1.29	0.42438
6	rs2239804	32411523	*HLA-DRA*	G	A	0.76	0.42	1.38	0.3706	0.307	0.3135	1.63	0.97	2.74	0.06782	0.4219	0.3151	1.17	0.79	1.73	0.43369
6	rs9471290	40260515	*LINC00951*	A	G	0.86	0.49	1.51	0.6037	0.339	0.3677	1.51	0.89	2.56	0.1297	0.4375	0.357	1.16	0.79	1.71	0.4541
6	rs9268528	32383108	*HLA-DRA*	G	A	0.65	0.35	1.21	0.1734	0.274	0.3187	1.72	1.03	2.86	0.03818	0.4375	0.3217	1.15	0.78	1.71	0.47132
6	rs6458146	40218128	*LINC00951*	G	A	0.71	0.39	1.27	0.2472	0.274	0.3341	1.18	0.68	2.05	0.5547	0.3438	0.3192	0.93	0.62	1.39	0.71688
6	rs2395163	32387809	*HLA-DRA*	G	A	1	0.52	1.92	0.9998	0.226	0.1865	0.95	0.49	1.85	0.8772	0.1719	0.1888	0.97	0.61	1.55	0.91381

Abbreviations: ASPD, antisocial personality disorder; BP, base pairs; GWAS, genome-wide association study; MAF, minor allele frequency; OR, odds ratio; SNP, single-nucleotide polymorphism.

In the total study population (meta-analysis), rs4714329 reached genome-wide significance (*P*=1.7 × 10^−9^).

## Supplementary Material

Supplementary Informations
