# Supplementary material for: Genome-wide association study of antisocial personality disorder
Source: Transl Psychiatry. 2016 Sep 6;6(9):e883–. doi: 10.1038/tp.2016.155 (PMC5048197; doi:10.1038/tp.2016.155)

**Supplementary Information**

**Genome-wide association study of antisocial personality disorder**

Marja-Riitta Rautiainen^1,2,3^, Tiina Paunio^1,3,4^*, Eila Repo-Tiihonen^2^, Matti Virkkunen^3^, Hanna M Ollila^1,5^, Sonja Sulkava^1,3^, Otto Jolanki^5^, Aarno Palotie^4,6,7,8,9^ and Jari Tiihonen^2,10^*

^1^ National Institute for Health and Welfare, Department of Health, Helsinki, Finland;

^2^ University of Eastern Finland, Department of Forensic Psychiatry, Niuvanniemi Hospital, Kuopio, Finland;

^3^ University of Helsinki and Helsinki University Hospital, Department of Psychiatry, Helsinki, Finland;

^4^ University of Helsinki, Institute for Molecular Medicine Finland, Helsinki, Finland;

^5^Stanford University Center for Sleep Sciences, Palo Alto, CA, USA;

^6^ Wellcome Trust Sanger Institute, Hinxton, Cambridgeshire, England;

^7^ Analytic and Translational Genetics Unit, Department of Medicine, Massachusetts General Hospital, Boston, MA, USA;

^8^ Program in Medical and Population Genetics, Broad Institute of MIT and Harvard, Cambridge, MA, USA;

^9^ Psychiatric & Neurodevelopmental Genetics Unit, Department of Psychiatry, Massachusetts General Hospital, Boston, MA, USA;

^10^ Karolinska Institutet, Department of Clinical Neuroscience, Stockholm, Sweden

Address correspondence to: Jari Tiihonen, MD, PhD, Karolinska Institutet, Department of Clinical Neuroscience, Byggnad R5, S-171 76 Stockholm, Sweden. [jari.tiihonen@ki.se](mailto:jari.tiihonen@ki.se)

or Tiina Paunio, MD, PhD, National Institute for Health and Welfare, Department of Health, PO Box 30, FI-00271 Helsinki, Finland, [tiina.paunio@thl.fi](mailto:tiina.paunio@thl.fi)

The Supplementary Information contains detailed information on the control cohorts of the study and the secondary phenotypes, as well as genotyping, quality control, statistical analyses, linkage disequilibrium (LD) examination, and the investigation of the correlations between variant genotype and gene expression levels in the brain and the testis tissues, and the additional study related tables and figures.

Table of Contents

[Supplementary Text 4](#__RefHeading___Toc449610122)

[The control cohorts of the study 4](#__RefHeading___Toc449610123)

[Individual SCID II items of ASPD 4](#__RefHeading___Toc449610124)

[Phenotype in the secondary analyses of the population-based sample 5](#__RefHeading___Toc449610125)

[Genotyping and quality control of the GWAS data 5](#__RefHeading___Toc449610126)

[Genotyping 5](#__RefHeading___Toc449610127)

[Quality Control 5](#__RefHeading___Toc449610128)

[HLA imputation of the GWAS data. 6](#__RefHeading___Toc449610129)

[Genotyping and quality control with Sequenom MassArray 6](#__RefHeading___Toc449610130)

[Re-genotyping 6](#__RefHeading___Toc449610131)

[Genotyping of the replication sample 7](#__RefHeading___Toc449610132)

[Quality Control 7](#__RefHeading___Toc449610133)

[Statistical analyses 7](#__RefHeading___Toc449610134)

[Linkage disequilibrium analyses of rs4714329 8](#__RefHeading___Toc449610135)

[GTEx Portal and Braineac database investigation on variant genotype correlation with gene expression 9](#__RefHeading___Toc449610136)

[GTEx Portal 9](#__RefHeading___Toc449610137)

[The Braineac database 9](#__RefHeading___Toc449610138)

[Supplementary Information References 10](#__RefHeading___Toc449610139)

[Supplementary Tables 12](#__RefHeading___Toc449610140)

[Supplementary Table 1a. The 50 most significant associations in the analysis of ASPD in the whole GWAS sample of ASPD cases compared with population based controls. 12](#__RefHeading___Toc449610141)

[Supplementary Table 1b. The 50 most significant associations in the analysis of ASPD in the male GWAS sub-sample of ASPD cases compared with population based male controls. 13](#__RefHeading___Toc449610142)

[Supplementary Table 2. Association of HLA-alleles with ASPD 15](#__RefHeading___Toc449610143)

[Supplementary Table 3. DRB1 alleles, amino acids and HLA-SNPs with P<0.0001 20](#__RefHeading___Toc449610144)

[Supplementary Table 4. The minor allele frequencies of the eight variants included in the replication analyses in HapMap-CEU, GWAS, and replication samples. 21](#__RefHeading___Toc449610145)

[Supplementary Table 5 a. The linkage disequilibrium (LD) in the CRIME and the 1000 Genomes samples between rs1471329 and variants within the nearby genes (RP11-552E20.1, TDRG1, LINC00951, and LRFN2). 22](#__RefHeading___Toc449610146)

[Supplementary Table 5 b. The linkage disequilibrium (LD) in the HapMap3 and in the 1000 Genomes samples between rs4714329 and variants within the nearby genes (RP11-552E20.1, TDRG1, LINC00951, and LRFN2). 22](#__RefHeading___Toc449610147)

[Supplementary Table 6 a. Results from the GTEx Portal investigation of rs4714329 association with TDRG1, LINC00951, and LRFN2 genes expression in brain and testis tissues. 24](#__RefHeading___Toc449610148)

[Supplementary Table 6 b. The ten most significant associations from the Braineac database investigation of rs4714329 association with gene expression in ten brain tissues. 25](#__RefHeading___Toc449610149)

[Supplementary Table 7. SCID-II questions and the corresponding item numbers and SCID-II question numbers. 26](#__RefHeading___Toc449610150)

[Supplementary Table 8. Results from the 22 individual SCID-II items. 27](#__RefHeading___Toc449610151)

[Supplementary Figures 29](#__RefHeading___Toc449610152)

[Supplementary Figure 1 a. Q-Q Plot of the association analysis of the combined sample of males and females. 29](#__RefHeading___Toc449610153)

[Supplementary Figure 1 b. Q-Q Plot of the association analysis of the male sub-sample. 30](#__RefHeading___Toc449610154)

[Supplementary Figure 2 a. Regional Manhattan Plot of chromosome 6 of the analysis of the combined sample of males and females. 31](#__RefHeading___Toc449610155)

[Supplementary Figure 2 b. Regional Manhattan Plot of chromosome 6 of the male sub-sample. 31](#__RefHeading___Toc449610156)

[Supplementary Figure 3. The Linkage Disequilibrium (LD) structure of the CRIME sample for rs4714329 and the other three selected SNPs on 6p21.2. 32](#__RefHeading___Toc449610157)

[Supplementary Figure 4 a. Rs4714329 linkage disequilibrium (LD) with the two SNPs of LINC00951 gene available in the CRIME sample. 33](#__RefHeading___Toc449610158)

[Supplementary Figure 4 b. Rs4714329 linkage disequilibrium (LD) with the 23 SNPs of LINC00951 gene SNPs available in the HapMap3 sample. 33](#__RefHeading___Toc449610159)

[Supplementary Figure 5. Results from the analysis of the 22 individual SCID-II items. 34](#__RefHeading___Toc449610160)

Supplementary Text

*The control cohorts of the study*

Health 2000 control cohort. The Health 2000 study is a Finnish nationwide survey carried out by the National Institute for Health and Welfare in 2000–2001 in order to investigate public health in Finland (http://www.terveys2000.fi/doc/methodologyrep.pdf). The GenMets study of metabolic syndrome is a sub-study of the Health 2000, where half of the participants have a metabolic syndrome, and the other half represent their age- and gender-matched controls.^1^ The GenMets subpopulation (N=2124) was used as a control group in the GWAS analyses in the present study. The remaining sample of Health 2000 (Health 2000 sample excluding GenMets subcohort, N=4476) was used as controls in the subsequent replication analysis. Following the discovery analyses and replication, the entire Health 2000 cohort (N=6600) was further used as a study cohort in the candidate polymorphism analyses in the general population. A written informed consent was obtained from participants. This part of the study was approved by the ethics committee of the Helsinki University Central Hospital.

FINRISK and Corogene control cohorts. The National FINRISK Study is a quinquennial nationwide survey carried out since 1972 to assess the risk factors of chronic diseases and health behavior in the working age population in Finland. DNA sample collection was included in the studies in 1987.^2^ The Corogene study is a sub-study of FINRISK including 5000 consecutive patients of Finnish origin assigned to coronary angiogram in the region of Helsinki University Central Hospital. Of the Corogene cohort, 2500 patients and an equal number of age-, geographical area- and sex-matched controls from the FINRISK surveys, underwent genome-wide genotyping.^3^ In the current study, the Corogene sample was used as controls in the genome-wide association analyses.

*Individual SCID II items of ASPD*

The analysis of individual SCID II items included the following items (items 1-13 concern the time before the age of 15; 14-1 before the age of 13; 16-22 after the age of 15): 1. “Often bullied, threatened, or intimidated others”; 2. “Often initiated physical fights”; 3. “Has used a weapon that can cause serious physical harm to others (e.g., a bat, brick, broken bottle, knife, gun)”; 4. “Has been physically cruel to people”; 5. “Has been physically cruel to animals”; 6. “Has stolen while confronting a victim (e.g., mugging, purse snatching, extortion, armed robbery)”; 7. “Has forced someone into sexual activity”; 8. “Has deliberately engaged in fire setting with the intention of causing serious damage”; 9. “Has deliberately destroyed other’s property (other than by fire setting)”; 10. “Has broken into someone else’s house, building, or car”; 11. “Often lies to obtain goods or favors or to avoid obligations (i.e., “cons” others)”; 12. “Has stolen items of nontrivial value without confronting victim (e.g., shoplifting, stealing but without breaking and entering, forgery)”; 13. “Has run away from home overnight at least twice while living in parental or parental surrogate home (or once without returning for a lengthy period)”; 14. “Often stayed out at night despite parental prohibitions”; 15. “Often truant from school”; 16. “Failure to conform to social norms with respect to lawful behaviours, as indicated by repeatedly performing acts that are ground for arrest”; 17. “Deceitfulness, as indicated by repeated lying, use of aliases, or conning others for personal profit or pleasure”; 18. “Impulsivity or failure to plan ahead”; 19. “Irritability and aggressiveness, as indicated by repeated physical fights or assaults”; 20. “Reckless disregard for safety of self or others”; 21. “Consistent irresponsibility, as indicated by repeated failure to sustain consistent work behavior or honor financial obligations”; 22. “Lacks remorse as indicated by being indifferent to, or rationalizing having hurt, mistreated or stolen from another”. The possible criterion statements in the questionnaire were “?” = “Inadequate information”, “1” = “Absent or False”, “2” = “Subthreshold”, and “3” = “Threshold or True”. Individuals with the item statement of “3” were included as cases of each item. The resulting subgroups overlapped partly.

*Phenotype in the secondary analyses of the population-based sample*

The following eight questions were utilized to achieve a measure for antisocial features in the population-based sample of Health 2000: 1. “I think most people would lie for their own benefit”; 2. “Most people are honest and honorable because of fear of getting caught”; 3. “In order to benefit, most people are ready to use dishonest means if honest ones do not help”; 4. “I commonly wonder what hidden reason another person may have for doing something for my benefit”; 5. “No one really cares what happens to others”; 6. “It is better not to trust anyone”; 7. “Most people make friends because friends are likely to be useful to them”; and 8. “Most people do not actually want to put themselves out to help others”. The possible response options were 1 = “Completely incorrect”, 2 = “Mostly incorrect”, 3 = “Mostly correct”, and 4 = “Completely correct”. This short scale has been previously utilized to measure the cognitive component of hostility.^4^ The childhood environment of economic difficulties or severe conflicts in the family were assessed with the questions “Did your childhood family have long-lasting financial problems?” and “Were there severe conflicts in your childhood family?”. The response options were “Yes”, “No”, and “I don’t know”. Participants reporting “I don’t know” were excluded from the analyses.

*Genotyping and quality control of the GWAS data*

**Genotyping**

In the CRIME cohort, DNA was extracted from peripheral blood leukocytes or saliva using the commercially-available Puregene DNA isolation kit (Puregene, Gentra Systems, Minneapolis, USA) and Autopure LS equipment (Qiagen, Crawley, UK).

CRIME cohort, comprising 579 individuals, was genotyped at the Welcome Trust Sanger Institute, Cambridge, UK, utilizing Illumina Human670-QuadCustom BeadChip, which assayed 594,398 common single nucleotide polymorphisms (SNPs). The GenMets and Corogene control samples were both genotyped with HumanHap610-Quad SNP array (Illumina Inc, San Diego, USA) at the Cambridge Welcome Trust Sanger Institute.

**Quality Control**

The quality control of the data was conducted utilizing PLINK v1.07, <http://pngu.mgh.harvard.edu/purcell/plink/>)^5^ and SPSS (IBM Corp. Released 2011. IBM SPSS Statistics for Windows, Version 20.0. Armonk, NY: IBM Corp.).

Altogether, 29 samples failed the Sanger quality control of CRIME cohort, and were removed from the data. We first conducted identical quality control for each three data sets included in the genome-wide analyses (CRIME cohort, GenMets control cohort, and CoroGene control cohort). As the GWAS study cases of the CRIME sample and the controls of the GenMets and Corogene samples were genotyped with different platforms (CRIME with Illumina Human670-QuadCustom BeadChip and GenMets and Corogene with Illumina Human610 Quad BeadChip), we applied more stringent criteria than usual in the quality control: For both samples and SNPs, the call rate threshold was > 99%. No sex discrepancies were observed in the examination of heterozygote rates of X-chromosomes. The genome-wide identity-by-descent (IBD) calculations were performed to identify relatedness and individuals that shared more than 20% of their genome were excluded. The genome-wide identity-by-state (IBS) estimation was also performed and population outliers were removed. For SNPs, the threshold for MAF was > 0.05, and HWE test p < 0.001. To avoid any ambiguity due to strand origins of the SNPs, all AT/TA and CG/GC SNPs were removed. The three datasets were then merged. Because of the different arrays utilized in the genotyping of the cases and the controls, only those SNPs that were originally included in all three data sets were kept in the merged data (at this point, altogether 481870 SNPs were overlapping all three datasets). A new quality control was performed for the merged dataset with the extremely stringent procedure, as in the initial QC that was performed for each individual data set. Again, population outliers were stringently removed according to the MDS clustering (138 individuals), and again, those individuals of a pair sharing more than 20% (pi-hat > 0.2) of their genome with less successful variant genotypes were removed (29 individuals). Altogether, 370 criminal offenders with ASPD and 5850 population controls, as well as 481866 SNPs, were included in the final dataset.

*HLA imputation of the GWAS data***.**

Imputation of the GWAS genotype data with the classical HLA-alleles was performed utilizing the HIBAG software^6^. HIBAG uses an attributed bagging algorithm, that estimates the HLA-alleles by averaging HLA-type posterior probabilities over an ensemble of classifiers (SNPs) built on bootstrap samples.^6^ The HLARES data, with 2517 individuals with both HLA typing and GWAS data, was used as a reference set. These data comprise European ancestry individuals from clinical studies conducted by GlaxoSmithKline over most European countries and populations.^7^ As we also examined the effect of non-synonymous mutations on ASPD, the amino acid polymorphisms from exon 2, which is the peptide binding region in HLA, were implemented using IMGT database version 3.14.0 (<http://www.imgt.org/>) utilizinging R version 3.0.2.^8^ Previous HLA typing studies have shown that individuals from Finland have unique HLA-haplotypes that are rare in other European populations. These haplotypes, such as A*03:01-B*35:01-DRB1*01:01, are often demanding to impute.^9,10^ As part of the imputation procedure, the imputation accuracy was estimated utilizing the known haplotype frequency of the A*03:01-B*35:01-DRB1*01:01-haplotype. The observed frequency was similar to that reported earlier (f typing = 0.071, f imputed controls = 0.082).^9,10^

*Genotyping and quality control with Sequenom MassArray*

**Re-genotyping**

In the GWAS study, the CRIME sample was genotyped with a different array (Illumina Human670-QuadCustom BeadChip) than the control samples, GenMets and Corogene (Illumina Human610 Quad BeadChip), although in the same facility of the Welcome Trust Sanger Institute (Cambridge, England). To examine the genotyping consistency, we re-genotyped the eight replication variants in CRIME samples (N = 493, including also non-ASPD samples) and GenMets samples (1800 samples of 2124 were available), that were included in the GWAS, utilizing Sequenom MassArray (IPlex) technology (Sequenom, San Diego, California). We then compared the original GWAS genotypes with the genotypes achieved with the Sequenom MassArray. The genotypes were over 99% similar in the GWAS sample and in the regenotyping.

**Genotyping of the replication sample**

Based on the GWAS results, we selected eight variants for replication genotyping in the samples of the CRIME cohort that were not included in the GWAS analysis (N = 182), and in the Health 2000 sample (N = 4476) excluding GenMets sub cohort that was used in the GWAS analysis as one of the control cohorts. The genotyping was conducted utilizing Sequenom MassArray (IPlex) technology (Sequenom, San Diego, California).

**Quality Control**

In the Sequenom genotyping, the thresholds for inclusion of samples and SNPs were as follows: for both samples and SNPs the genotype call rate was > 0.95, for the SNPs MAF > 0.01, and HWE test P < 10^-6^. After the QC, there remained 173 CRIME cases with antisocial personality disorder and 3766 Health 2000 controls (GenMets subcohort excluded) (Table 1) and all of the SNPs passed the QC.

*Statistical analyses*

All of the association analyses were performed using a generalized linear (logistic) model with PLINK v1.07 with age and sex and the ten first MDS clusters as covariates. The analyses were performed in the entire group of individuals with antisocial personality diagnosis compared with population controls, as well as in the male sub-sample separately. The number of the female participants (31) was insufficient for a separate analysis. The meta-analyses with fixed-effect model were performed with GWAMA software (http://www.geenivaramu.ee/en/tools/gwama.^11^ SPSS (IBM Corp. Released 2013. IBM SPSS Statistics for Windows, Version 22.0. Armonk, NY: IBM Corp.) was utilized in the gene x environment analysis to assess a generalized linear model including an interaction term.

The Haploview^12^ was used for creating the Manhattan Plots of all chromosomes and of the entire sample of males and females combined in the analysis of the ASPD cases versus population-based controls and for the males’ sub-sample (Figure 2 a and b), as well as for the regional plots of chr 6 (Supplementary Figure 2 a and b). The regional plots of (entire sample) the variants on 6p21.2, near rs4714329 (Figure 3 a), and the variants on 6p21.32, near rs9268528 (Figure 3 b), were created with LocusZoom.^13^ The quantile-quantile (Q-Q) plots of the association analysis of the combined sample of males and females (Supplementary Figure 1 a) and the male sub-sample (Supplementary Figure 1 b) were created utilizing the qqman package in R.^14^

*Power calculations.* The power calculations were performed utilizing Genetic Power Calculator (<http://pngu.mgh.harvard.edu/~purcell/gpc/>).^15^ The analysis of males and females combined had 87.8% power (alpha = 0.05) to detect variants with a MAF of 0.4 (assuming additive model, genotype relative risk 1.3, and a perfect linkage disequilibrium between the trait and locus and a marker). However, with an alpha of 5x10^-8^, the power decreases to 1% and for 80% power, the required number of cases would be 1502. The analysis of males demonstrated only 83.5% power with the alpha of 0.05, and 0.6% with the alpha of 5x10^-8^. The power for the replication of rs4714329 in the replication sample of CRIME subjects those were not included in the GWAS analysis was 87.9% in the analysis of males and females combined (alpha = 0.05, MAF = 0.443, additive model, genotype relative risk 1.5, and a perfect linkage disequilibrium between the trait, the locus and the marker), and the analysis of males demonstrated only 88.1% power for replication (MAF = 0.445, genotype relative risk 1.57).

*Linkage disequilibrium analyses of rs4714329*

The linkage disequilibrium (LD) and haplotype analyses were performed utilizing PLINK v1.07 (utilizing CRIME sample genotypes, and cases and controls included in ASPD analysis, respectively), Haploview ^12^ (in the LD analysis, utilizing CRIME sample and HapMap3 genotypes), and SNAP^16^ (in the LD analysis, utilizing 1000 Genomes Pilot 1). Haploview was also utilized for the visualization of the haploblocks and the LD structure of the CRIME sample for rs4714329 and the other three selected SNPs on 6p21.2 (Supplementary Figure 3), for the LD of rs4714329 with the two SNPs of LINC00951 gene available in the CRIME sample (Supplementary Figure 4 a) and with the 23 SNPs of LINC00951 gene SNPs available in the HapMap3 sample (Supplementary Figure 4 b).

Rs4714329 resides in an intergenic region on chr 6p21.2. in the vicinity of non-protein coding genes *RP11-552E20.1* (ENSG00000227131, chr6:40,271,566-40,276,237, reverse strand, GRCh38/hg38), *Testis Development Related 1* (*TDRG1*, ENSG00000204091, chr6:40,334,954-40,379,887, GRCh38/hg38, forward strand), and *Long Intergenic Non-Protein Coding RNA 951* (*LINC00951*, ENSG00000204092, chr6: 40,344,344-40,346,151, GRCh38/hg38, reverse strand). The nearest protein coding gene is *Leucine Rich Repeat and Fibronectin Type III Domain Containing 2* (*LRFN2*, ENSG00000156564, chr6: 40,391,586-40,587,465, reverse strand) gene. The CRIME data included two variants of the *LINC00951* gene, three variants of the *TDRG1* gene, and 50 of the *LRFN2* gene. No variants were available of the *RP11-552E20.1* gene. None of the studied variants were in complete LD with rs4714329, however, a relatively strong LD was observed with the two available LINC00951 variants rs17619142 (D’ = 0.872, LOD = 34.55, r^2^ = 0.255) and rs17619309 (D’ = 0.897, LOD = 25.06, r^2^ = 0.178) (Supplementary Table 5a, Supplementary Figure 4a). In the GWAS analysis, the same variants of *LINC00951* gene revealed nominal association (ORs of 1.18 and 1.5, and P values of 0.002 and 0.159, respectively). Only a weak LD was observed with all the three available *TDRG1* variants as well as with several variants of the *LRFN2* gene (Supplementary Table 5a), however, one *LRFN2* gene variant, rs6925172, was also nominally associated with ASPD in the GWAS (OR = 1.36, P = 0.035). In the HapMap3 data, there were ten variants available of *RP11-552E20.1*, 23 of *LINC00951*, 13 of *TDRG1*, and 294 of *LRFN2*. The rs4714329 variant was consistently in complete LD with two variants, rs2504824 and rs12206539, and in a strong LD with several variants, of the *LINC00951* gene (Supplementary Table 5 b and Supplementary Figure 4 b). Only nominal LD was observed with one variant of *RP11-552E20.1*, rs9394677, and no LD was observed with any of the *LRFN2* variants. The SNAP investigation utilizing 1000 genomes Pilot 1 data for the nearby resident genes consistently revealed the strongest LD for variants (72 SNPs) in the *LINC00951* gene (r^2^ range 0.341-0.003). For *TDRG1* gene (20 SNPs), the r^2^ ranged from 0.06 to 0, and for the *LRFN2* gene (808 SNPs) from 0.128 to 0. Thus, the LD investigation results from the CRIME as well as the HapMap3 and 1000 Genomes samples indicate that *LINC00951* is the strongest candidate of the nearby genes for the signal origin.

*GTEx Portal and Braineac database investigation on variant genotype correlation with gene expression*

In this study, the GTEx Portal (<http://www.gtexportal.org/home/>) and the Braineac - The Brain eQTL Almanac (<http://www.braineac.org/>) were utilized to test our most significant variant, rs4714329, for association with genes residing nearby (*LRFN2* (ENSG00000156564.8), *LINC00951* (ENSG00000204092.2 and *TDRG1* (ENSG00000204091.3)). Expression was measured in all of the brain tissues and testis tissue available in GTEx (brain - amygdala (N = 62), brain - anterior cingulate cortex (N = 72), brain - caudate (basal ganglia) (N = 100), brain - cerebellar hemisphere (N = 89), brain - cerebellum (N = 103), brain - cortex (N = 96), brain - frontal cortex (N = 92), brain - hippocampus (N = 81), brain - hypothalamus (N = 81), brain - nucleus accumbens (N = 72), brain - putamen (N = 82), brain - spinal cord (N = 59), brain - substantia nigra (N = 56), and testis (N = 157). In the Braineac, expression was measured in the cerebellar cortex (CRBL, N = 130), frontal cortex (FCTX, N = 127), hippocampus (HIPP, N = 122), medulla (specifically inferior olivary nucleus, MEDU, N = 119), occipital cortex (specifically primary visual cortex, OCTX, N = 129), putamen (PUTM, N = 129), substantia nigra (SNIG, N = 101), thalamus (THAL, N = 124), temporal cortex (TCTX, N = 119) and intralobular white matter (WHMT, N = 131). No data was available for the RP11-552E20.1 gene (ENSG00000227131.1) in the GTEx Portal, and for RP11-552E20.1 and LINC00951 genes in the Braineac.

**GTEx Portal**

The Genotype-Tissue Expression (GTEx) Portal is a resource database and associated tissue bank designed for investigations into the relationship between genetic variation and gene expression in a variety of human tissues. The tissues are collected from autopsies (within 24 h of death) or organ and tissue transplantations and surgeries. Peripheral blood samples are also collected for genotyping and to establish lymphoblastoid cell lines and skin samples are collected for measurement of gene expression and to establish fibroblast cultures. The tissues are typically collected from many predesignated tissue sites and organs of each donor. Eligible donors may be of either sex and between 21 and 70 years of age, however, individuals carrying human immunodeficiency virus (HIV) or of high-risk behaviors, viral hepatitis, metastatic cancer, chemotherapy, or radiation therapy within the past 2 years, whole blood transfusion in the past 48h, or a body mass index of >35 of <18.5 are excluded. Brain donors must not have been on a ventilator within 24 h prior to death. DNA genotyping is performed from each donor’s blood sample (or an alternate tissue, when blood is unavailable), and RNA is sequenced from each tissue.^17^

**The Braineac database**

The Braineac - The Brain eQTL Almanac is a resource database to access the UK Brain Expression Consortium (UKBEC) dataset. The aim of Braineac is to release to the scientific community a valid instrument to investigate the genes and SNPs associated with neurological disorders. With the Braineac, it possible to visualise gene expression across the brain, to find out whether a SNP of interest is acting as cis-eQTL on a selected gene and know where it's located, to determine whether one or more risk SNPs operate as an eQTL and in which brain region, and to download the expression data by genes or tissue. The dataset currently comprises 134 brains, collected by the Medical Research Council (MRC) Sudden Death Brain and Tissue Bank, Edinburgh, UK ^18^, and the Sun Health Research Institute (SHRI) an affiliate of Sun Health Corporation, USA.^19^ All individuals were confirmed to be neuropathologically normal by a consultant neuropathologist using histology performed on sections prepared from paraffin-embedded brain tissue blocks. The brain regions were extracted from each brain in parallel for mRNA quantification. From each individual up to 10 brain regions were analyzed. DNA was collected to enable genotyping and expression quantitative trait loci (eQTL) analysis. The DNA was extracted from sub-dissected samples (100–200 mg) of human post-mortem brain tissue. Total RNA was also isolated from the post-mortem brain tissues.^20^

Supplementary Information References

1. Kristiansson K, Perola M, Tikkanen E, Kettunen J, Surakka I, Havulinna AS *et al.* Genome-wide screen for metabolic syndrome susceptibility Loci reveals strong lipid gene contribution but no evidence for common genetic basis for clustering of metabolic syndrome traits. *Circ Cardiovasc Genet* 2012; **5:** 242–9.

2. Vartiainen E, Jousilahti P, Alfthan G, Sundvall J, Pietinen P, Puska P. Cardiovascular risk factor changes in Finland, 1972–1997. *Int J Epidemiol* 2000; **29:** 49–56.

3. Vaara S, Nieminen MS, Lokki ML, Perola M, Pussinen PJ, Allonen J *et al.* Cohort profile: the Corogene study. *Int J Epidemiol* 2012; **41:** 1265–71.

4. Greenglass ER, Julkunen J. Construct validity and sex differences in Cook‐Medley hostility. *Pers* *Individ* *Dif* 1989; **10:** 209–18.

5. Purcell S, Neale B, Todd-Brown K, Thomas L, Ferreira MA, Bender D *et al.* PLINK: a tool set for whole-genome association and population-based linkage analysis. *Am* *J Hum Genet* 2007; **81:** 559–75.

6. Zheng X, Shen J, Cox C, Wakefield JC, Ehm MG, Nelson MR *et al.* HIBAG--HLA genotype imputation with attribute bagging. *Pharmacogenomics J* 2014; **14:** 192–200.

7. Dilthey A, Leslie S, Moutsianas L, Shen J, Cox C, Nelson MR *et al.* Multi-population classical HLA type imputation. *PLoS Comput Biol* 2013; **9:** e1002877.

8. R Development Core Team. R: A language and environment for statistical computing. R Foundation for Statistical Computing, Vienna, Austria. Retrieved from [http://www.R-project.org](http://www.R-project.org/), 2008.

9. Wennerström A, Vlachopoulou E, Lahtela LE, Paakkanen R, Eronen KT, Seppänen M *et al.* Diversity of extended HLA-DRB1 haplotypes in the Finnish population. *PLoS One* 2013; **8:** e79690.

10. Vlachopoulou E, Lahtela E, Wennerström A, Havulinna AS, Salo P, Perola M *et al.* Evaluation of HLA-DRB1 imputation using a Finnish dataset. *Tissue Antigens* 2014; **83:** 350–5.

11. Mägi R, Morris AP. GWAMA: software for genome-wide association meta-analysis. BMC *Bioinformatics* 2010; **11:** 288.

12. Barrett JC, Fry B, Maller J, Daly MJ. Haploview: analysis and visualization of LD and haplotype maps. *Bioinformatics* 2005; **21:** 263–6.

13. Pruim RJ*, Welch RP*, Sanna S, Teslovich TM, Chines PS, Gliedt TP, Boehnke M, Abecasis GR, Willer CJ. (2010) LocusZoom: Regional visualization of genome-wide association scan results. *Bioinformatics* 2010 September 15; 26(18): 2336.2337.

14. Turner, S.D. qqman: an R package for visualizing GWAS results using Q-Q and manhattan plots. biorXiv DOI: 10.1101/005165.

15. Purcell S, Cherny SS, Sham PC. Genetic Power Calculator: design of linkage and association genetic mapping studies of complex traits. *Bioinformatics* 2003; **19:** 149–50.

16. Johnson, A. D., Handsaker, R. E., Pulit, S., Nizzari, M. M., O'Donnell, C. J., de Bakker, P. I. W. SNAP: A web-based tool for identification and annotation of proxy SNPs using HapMap

17. GTEx Consortium. The Genotype-Tissue Expression (GTEx) project. *Nat Genet* 2013; **45:** 580–5.

18. Millar T, Walker R, Arango JC, Ironside JW, Harrison DJ, MacIntyre DJ, *et al.* Tissue and organ donation for research in forensic pathology: the MRC Sudden Death Brain and Tissue Bank. *J Pathol.* 2007 Dec;213(4):369-75.

19. Beach TG, Sue LI, Walker DG, Roher AE, Lue L, Vedders L, *et al.* The Sun Health Research Institute Brain Donation Program: Description and Eexperience, 1987–2007. *Cell Tissue Bank.* 2008; 9(3):229-45.

20. Ramasamy A, Trabzuni D, Guelfi S, Varghese V, Smith C, Walker R, et al. Genetic variability in the regulation of gene expression in ten regions of the human brain. Nat Neurosci. 2014;17(10):1418–28. doi: 10.1038/nn.3801 pmid:25174004.

Supplementary Tables

Supplementary Table 1a. The 50 most significant associations in the analysis of ASPD in the whole GWAS sample of ASPD cases compared with population-based controls.

| **CHR** | **SNP** | **BP** | **A1** | **A2** | **OR** | **L95** | **U95** | **P** | **MAF CASES** | **MAF CTRLS** | **GENE (OR**  **NEAREST GENE)** |
| --- | --- | --- | --- | --- | --- | --- | --- | --- | --- | --- | --- |
|
| 7 | rs6462756 | 4338311 | A | C | 1.839 | 1.449 | 2.333 | 5.47E-07 | 0.2302 | 0.1459 | (SDK1) |
| 6 | rs9268528 | 32491086 | G | A | 0.576 | 0.462 | 0.719 | 9.89E-07 | 0.2486 | 0.3296 | (BTNL2/HLA-DRA) |
| 6 | rs9268542 | 32492699 | G | A | 0.578 | 0.463 | 0.721 | 1.12E-06 | 0.2500 | 0.3307 | (BTNL2/HLA-DRA) |
| 1 | rs10914134 | 179158165 | A | C | 1.618 | 1.317 | 1.989 | 4.74E-06 | 0.3108 | 0.2684 | KIAA1614 |
| 7 | rs12671939 | 52666021 | A | G | 0.517 | 0.389 | 0.688 | 5.55E-06 | 0.1135 | 0.1570 | (LOC101928257) |
| 7 | rs13243026 | 52701822 | A | G | 0.525 | 0.397 | 0.693 | 5.68E-06 | 0.1196 | 0.1651 | (LOC101928257) |
| 2 | rs6760287 | 238163735 | G | A | 1.819 | 1.404 | 2.355 | 5.76E-06 | 0.1757 | 0.1267 | RAB17 |
| 7 | rs1442256 | 52646539 | C | A | 0.539 | 0.412 | 0.705 | 6.87E-06 | 0.1311 | 0.1749 | (LOC101928257) |
| 6 | rs9471290 | 40368493 | A | G | 1.534 | 1.27 | 1.851 | 8.51E-06 | 0.4297 | 0.3667 | (LINC00951) |
| 7 | rs17135206 | 52623046 | G | A | 0.561 | 0.434 | 0.724 | 9.5E-06 | 0.1527 | 0.1950 | (LOC101928257) |
| 7 | rs2877031 | 52624891 | A | G | 0.561 | 0.434 | 0.724 | 9.55E-06 | 0.1527 | 0.1951 | (LOC101928257) |
| 7 | rs12718691 | 52642675 | G | A | 0.554 | 0.426 | 0.720 | 9.78E-06 | 0.1459 | 0.1849 | (LOC101928257) |
| 7 | rs2158044 | 93006335 | G | A | 0.634 | 0.517 | 0.777 | 1.1E-05 | 0.3014 | 0.3674 | CALCR |
| 7 | rs6973591 | 92998607 | G | A | 0.634 | 0.518 | 0.777 | 1.12E-05 | 0.3014 | 0.3672 | CALCR |
| 6 | rs2239804 | 32519501 | G | A | 0.613 | 0.493 | 0.763 | 1.19E-05 | 0.2500 | 0.3219 | HLA-DRA |
| 6 | rs6458146 | 40326106 | G | A | 1.528 | 1.263 | 1.850 | 1.32E-05 | 0.3932 | 0.3270 | (LINC00951) |
| 5 | rs332807 | 16553790 | G | A | 1.504 | 1.251 | 1.808 | 1.39E-05 | 0.5784 | 0.4907 | FAM134B |
| 7 | rs12670614 | 52675918 | A | G | 0.554 | 0.424 | 0.723 | 1.39E-05 | 0.1365 | 0.1752 | (LOC101928257) |
| 2 | rs283826 | 79086587 | A | G | 1.557 | 1.273 | 1.904 | 1.58E-05 | 0.3324 | 0.2728 | (REG3G) |
| 2 | rs393149 | 79097715 | G | A | 1.554 | 1.272 | 1.900 | 1.64E-05 | 0.3405 | 0.2815 | (REG3G) |
| 6 | rs9459805 | 167256141 | G | A | 1.747 | 1.355 | 2.252 | 1.65E-05 | 0.1797 | 0.1228 | (RNASET2) |
| 8 | rs1554057 | 66452141 | G | A | 1.812 | 1.382 | 2.374 | 1.65E-05 | 0.1622 | 0.1168 | (LINC01299) |
| 1 | rs2495090 | 14027642 | A | G | 1.624 | 1.302 | 2.024 | 1.68E-05 | 0.2779 | 0.2165 | (PRDM2) |
| 2 | rs283832 | 79090597 | G | A | 1.554 | 1.271 | 1.899 | 1.69E-05 | 0.3324 | 0.2731 | (REG3G) |
| 5 | rs40032 | 16561139 | G | A | 0.670 | 0.557 | 0.806 | 2.11E-05 | 0.4230 | 0.5038 | FAM134B |
| 6 | rs10498746 | 40332246 | G | A | 1.526 | 1.254 | 1.857 | 2.46E-05 | 0.3527 | 0.2809 | (LINC00951) |
| 11 | rs6591967 | 80807996 | A | G | 1.524 | 1.252 | 1.854 | 2.63E-05 | 0.3932 | 0.3315 | (LOC101928989) |
| 2 | rs12474421 | 202672432 | A | G | 1.515 | 1.248 | 1.840 | 2.75E-05 | 0.3527 | 0.2945 | LOC100652824 |
| 6 | rs4714329 | 40381435 | G | A | 1.500 | 1.241 | 1.814 | 2.78E-05 | 0.4432 | 0.3855 | (LINC00951) |
| 16 | rs12923860 | 72097466 | A | G | 2.049 | 1.461 | 2.874 | 3.2E-05 | 0.0973 | 0.0647 | LOC101927998 |
| 4 | rs2055212 | 143325156 | G | A | 1.649 | 1.301 | 2.090 | 3.49E-05 | 0.1946 | 0.1623 | INPP4B |
| 3 | rs9818348 | 54346185 | G | A | 0.673 | 0.557 | 0.813 | 4.11E-05 | 0.3649 | 0.4110 | CACNA2D3 |
| 3 | rs1703802 | 152230841 | A | C | 0.537 | 0.398 | 0.724 | 4.68E-05 | 0.0973 | 0.1440 | CLRN1-AS1 |
| 6 | rs9268615 | 32510867 | A | G | 0.634 | 0.510 | 0.790 | 4.78E-05 | 0.2486 | 0.3158 | (HLA-DRA) |
| 12 | rs2242384 | 75962567 | G | A | 1.523 | 1.243 | 1.867 | 4.91E-05 | 0.3027 | 0.2492 | E2F7 |
| 18 | rs4396629 | 63273244 | A | C | 1.924 | 1.401 | 2.640 | 5.16E-05 | 0.1095 | 0.0790 | (DSEL) |
| 13 | rs7337206 | 73628316 | G | A | 0.6 | 0.468 | 0.769 | 5.38E-05 | 0.1541 | 0.1972 | (KLF12) |
| 8 | rs6987649 | 42400791 | G | A | 1.676 | 1.303 | 2.157 | 5.78E-05 | 0.1676 | 0.1197 | SLC20A2 |
| 6 | rs3104405 | 32790286 | C | A | 1.525 | 1.241 | 1.874 | 5.91E-05 | 0.5216 | 0.4613 | (MTCO3P1) |
| 16 | rs205389 | 27957075 | A | G | 1.46 | 1.214 | 1.757 | 6.01E-05 | 0.5459 | 0.4845 | GSG1L |
| 15 | rs10152510 | 98206602 | C | A | 2.014 | 1.430 | 2.836 | 6.13E-05 | 0.0905 | 0.0612 | (DNM1P46) |
| 6 | rs2395163 | 32495787 | G | A | 0.592 | 0.458 | 0.765 | 6.23E-05 | 0.1365 | 0.1903 | (BTNL2/HLA-DRA) |
| 12 | rs17499173 | 115240447 | A | G | 1.638 | 1.286 | 2.087 | 6.32E-05 | 0.2041 | 0.1558 | (MED13L) |
| 9 | rs11787779 | 116781161 | A | G | 1.563 | 1.255 | 1.946 | 6.48E-05 | 0.2554 | 0.2001 | (TNC) |
| 6 | rs2395743 | 40400147 | G | A | 1.472 | 1.218 | 1.78 | 0.000065 | 0.4297 | 0.3719 | (LINC00951) |
| 6 | rs2395185 | 32541145 | A | C | 0.623 | 0.494 | 0.786 | 6.69E-05 | 0.1905 | 0.2447 | (HLA-DRB9) |
| 6 | rs1488 | 161458240 | G | A | 0.666 | 0.545 | 0.814 | 6.85E-05 | 0.2905 | 0.3739 | MAP3K4 |
| 15 | rs2727165 | 98220582 | C | A | 2.006 | 1.424 | 2.827 | 6.91E-05 | 0.0905 | 0.0611 | (DNM1P46) |
| 15 | rs2573632 | 98217286 | A | G | 2.006 | 1.424 | 2.827 | 6.95E-05 | 0.0905 | 0.0613 | (DNM1P46) |
| 5 | rs10039070 | 159915140 | C | A | 1.706 | 1.311 | 2.220 | 7.03E-05 | 0.1844 | 0.1077 | (ATP10B) |

Supplementary Table 1b. The 50 most significant associations in the analysis of ASPD in the male GWAS sub-sample of ASPD cases compared with population-based male controls.

| **CHR** | **SNP** | **BP** | **A1** | **A2** | **OR** | **SE** | **L95** | **P** | **MAF CASES** | **MAF CTRLS** | **GENE (OR NEAREST GENE)** |
| --- | --- | --- | --- | --- | --- | --- | --- | --- | --- | --- | --- |
|
| 6 | rs6458146 | 40326106 | G | A | 1.723 | 0.107 | 1.397 | 3.86E-07 | 0.4041 | 0.3217 | (LINC00951) |
| 6 | rs9268528 | 32491086 | G | A | 0.543 | 0.123 | 0.427 | 7.49E-07 | 0.2463 | 0.3377 | (BTNL2/HLA-DRA) |
| 6 | rs9471290 | 40368493 | A | G | 1.679 | 0.105 | 1.367 | 8.05E-07 | 0.4381 | 0.3660 | (LINC00951) |
| 6 | rs9268542 | 32492699 | G | A | 0.545 | 0.123 | 0.428 | 8.52E-07 | 0.2478 | 0.3392 | (BTNL2/HLA-DRA) |
| 6 | rs10498746 | 40332246 | G | A | 1.715 | 0.110 | 1.382 | 9.94E-07 | 0.3643 | 0.2760 | (LINC00951) |
| 7 | rs6462756 | 4338311 | A | C | 1.877 | 0.132 | 1.449 | 1.87E-06 | 0.2336 | 0.1462 | (SDK1) |
| 6 | rs7749170 | 40305179 | A | G | 1.673 | 0.111 | 1.345 | 3.76E-06 | 0.3466 | 0.2611 | (LINC00951) |
| 9 | rs1570479 | 648151 | G | A | 2.060 | 0.157 | 1.516 | 3.91E-06 | 0.1519 | 0.1109 | KANK1 |
| 6 | rs2239804 | 32519501 | G | A | 0.568 | 0.123 | 0.446 | 3.96E-06 | 0.2448 | 0.3283 | HLA-DRA |
| 13 | rs7337206 | 73628316 | G | A | 0.532 | 0.138 | 0.406 | 5.07E-06 | 0.1475 | 0.1970 | (KLF12) |
| 13 | rs1171092 | 35407728 | A | G | 0.546 | 0.133 | 0.420 | 5.43E-06 | 0.1726 | 0.2427 | DCLK1 |
| 6 | rs2395163 | 32495787 | G | A | 0.518 | 0.145 | 0.390 | 5.51E-06 | 0.1283 | 0.1931 | (BTNL2/HLA-DRA) |
| 13 | rs1171090 | 35408728 | A | G | 0.547 | 0.134 | 0.421 | 6.33E-06 | 0.1726 | 0.2426 | DCLK1 |
| 9 | rs9408655 | 649722 | A | G | 1.817 | 0.135 | 1.395 | 9.50E-06 | 0.2109 | 0.1682 | KANK1 |
| 9 | rs10815440 | 651057 | A | G | 1.810 | 0.135 | 1.389 | 1.09E-05 | 0.2109 | 0.1680 | KANK1 |
| 2 | rs12474421 | 203000000 | A | G | 1.608 | 0.109 | 1.298 | 1.36E-05 | 0.3555 | 0.2901 | LOC100652824 |
| 4 | rs2055212 | 143000000 | G | A | 1.791 | 0.134 | 1.377 | 1.42E-05 | 0.1917 | 0.1529 | INPP4B |
| 1 | rs10914134 | 179000000 | A | C | 1.644 | 0.116 | 1.311 | 1.70E-05 | 0.3068 | 0.2660 | KIAA1614 |
| 6 | rs9268615 | 32510867 | A | G | 0.591 | 0.123 | 0.465 | 1.88E-05 | 0.2434 | 0.3208 | (HLA-DRA) |
| 2 | rs974529 | 138000000 | G | A | 1.899 | 0.151 | 1.413 | 2.10E-05 | 0.1445 | 0.1116 | THSD7B |
| 6 | rs4714329 | 40381435 | G | A | 1.559 | 0.105 | 1.268 | 2.52E-05 | 0.4454 | 0.3833 | (LINC00951) |
| 2 | rs6760287 | 238000000 | G | A | 1.835 | 0.144 | 1.384 | 2.53E-05 | 0.1770 | 0.1304 | RAB17 |
| 14 | rs2065134 | 22328029 | C | A | 2.409 | 0.209 | 1.599 | 2.62E-05 | 0.0826 | 0.0554 | SLC7A7 |
| 7 | rs13243026 | 52701822 | A | G | 0.522 | 0.155 | 0.386 | 2.63E-05 | 0.1202 | 0.1619 | (LOC101928257) |
| 6 | rs2395175 | 32513004 | A | G | 0.503 | 0.164 | 0.364 | 2.82E-05 | 0.1018 | 0.1503 | (HLA-DRA) |
| 9 | rs1933793 | 103000000 | A | C | 1.701 | 0.127 | 1.326 | 2.86E-05 | 0.2183 | 0.1702 | (LOC644160) |
| 16 | rs12923860 | 72097466 | A | G | 2.241 | 0.193 | 1.534 | 2.99E-05 | 0.0959 | 0.0640 | LOC101927998 |
| 5 | rs936912 | 176000000 | C | A | 1.618 | 0.116 | 1.289 | 3.48E-05 | 0.3289 | 0.2735 | CDHR2 |
| 13 | rs7989245 | 35397597 | G | A | 0.602 | 0.123 | 0.473 | 3.72E-05 | 0.2109 | 0.2754 | DCLK1 |
| 7 | rs12671939 | 52666021 | A | G | 0.523 | 0.157 | 0.384 | 3.76E-05 | 0.1150 | 0.1545 | (LOC101928257) |
| 13 | rs9601689 | 35411429 | A | G | 0.608 | 0.121 | 0.480 | 3.86E-05 | 0.2198 | 0.2833 | DCLK1 |
| 9 | rs11787779 | 117000000 | A | G | 1.660 | 0.123 | 1.303 | 4.05E-05 | 0.2566 | 0.1983 | (TNC) |
| 7 | rs2158044 | 93006335 | G | A | 0.629 | 0.113 | 0.505 | 4.08E-05 | 0.3053 | 0.3680 | CALCR |
| 7 | rs6973591 | 92998607 | G | A | 0.630 | 0.113 | 0.505 | 4.15E-05 | 0.3053 | 0.3676 | CALCR |
| 4 | rs3775671 | 143000000 | A | G | 1.803 | 0.144 | 1.359 | 4.47E-05 | 0.1622 | 0.1272 | INPP4B |
| 5 | rs10039070 | 160000000 | C | A | 1.812 | 0.146 | 1.361 | 4.66E-05 | 0.1910 | 0.1096 | (ATP10B) |
| 7 | rs700273 | 146000000 | A | C | 0.643 | 0.109 | 0.519 | 4.75E-05 | 0.3127 | 0.3693 | CNTNAP2 |
| 6 | rs2395185 | 32541145 | A | C | 0.592 | 0.129 | 0.460 | 4.89E-05 | 0.1888 | 0.2489 | (HLA-DRB9) |
| 14 | rs2803953 | 72141256 | A | G | 2.155 | 0.189 | 1.487 | 4.96E-05 | 0.0915 | 0.0569 | (DPF3) |
| 13 | rs1936004 | 35419433 | A | C | 0.615 | 0.120 | 0.486 | 5.18E-05 | 0.2153 | 0.2802 | DCLK1 |
| 6 | rs2395743 | 40400147 | G | A | 1.529 | 0.106 | 1.243 | 5.74E-05 | 0.4322 | 0.3709 | (LINC00951) |
| 9 | rs10975792 | 670323 | A | G | 2.049 | 0.179 | 1.444 | 5.91E-05 | 0.1136 | 0.0893 | KANK1 |
| 1 | rs12121953 | 112000000 | A | G | 1.55 | 0.109 | 1.251 | 6.09E-05 | 0.3757 | 0.3164 | (LOC643355) |
| 1 | rs385768 | 112000000 | A | G | 1.549 | 0.109 | 1.251 | 6.16E-05 | 0.3761 | 0.3168 | (LOC643355) |
| 2 | rs1521674 | 82160306 | A | G | 2.066 | 0.181 | 1.449 | 6.18E-05 | 0.1003 | 0.0693 | (LYARP1) |
| 6 | rs1488 | 161000000 | G | A | 0.643 | 0.111 | 0.517 | 6.55E-05 | 0.2876 | 0.3775 | MAP3K4 |
| 8 | rs11993990 | 21601012 | G | A | 1.541 | 0.108 | 1.246 | 6.58E-05 | 0.3658 | 0.3116 | GFRA2 |
| 14 | rs17769369 | 39303108 | A | G | 1.674 | 0.130 | 1.299 | 6.90E-05 | 0.2434 | 0.1851 | (FBXO33) |
| 2 | rs4599058 | 236000000 | A | G | 0.595 | 0.131 | 0.460 | 6.91E-05 | 0.1770 | 0.2522 | (LOC642692) |
| 13 | rs4770705 | 24006315 | G | A | 0.64 | 0.112 | 0.513 | 6.93E-05 | 0.3142 | 0.3834 | (LOC101927375) |

Supplementary Table 2. Association of HLA-alleles with ASPD

| **CHR** | **Allele** | **OR** | **L95** | **U95** | **P** |
| --- | --- | --- | --- | --- | --- |
| 6 | DRB10101 | 2.19 | 1.53 | 3.141 | 1.91E-05 |
| 6 | DRB11302 | 1.27 | 0.759 | 2.131 | 0.3612 |
| 6 | DRB10301 | 1.35 | 0.8335 | 2.201 | 0.2207 |
| 6 | DRB10404 | 0.32 | 0.18 | 0.5672 | 9.75E-05 |
| 6 | DRB11001 | 0.15 | 0.01837 | 1.164 | 0.06933 |
| 6 | DRB11301 | 1.01 | 0.7162 | 1.435 | 0.9385 |
| 6 | DRB10701 | 0.95 | 0.6229 | 1.448 | 0.8101 |
| 6 | DRB10802 | 1.26 | 0.1543 | 10.25 | 0.8305 |
| 6 | DRB10801 | 1.01 | 0.7255 | 1.418 | 0.9339 |
| 6 | DRB11303 | 2.09 | 0.755 | 5.81 | 0.1556 |
| 6 | DRB10901 | 0.72 | 0.4094 | 1.275 | 0.2622 |
| 6 | DRB10401 | 0.66 | 0.4539 | 0.9559 | 0.02801 |
| 6 | DRB11501 | 1.40 | 1.038 | 1.869 | 0.02717 |
| 6 | DRB11101 | 0.87 | 0.5388 | 1.405 | 0.5688 |
| 6 | DRB10407 | 0.20 | 0.0237 | 1.706 | 0.1414 |
| 6 | DRB11401 | 1.44 | 0.5332 | 3.901 | 0.4708 |
| 6 | DRB11201 | 0.67 | 0.3621 | 1.254 | 0.2128 |
| 6 | DRB10408 | 1.26 | 0.1216 | 12.96 | 0.8485 |
| 6 | DRB10102 | 1.87 | 0.3193 | 10.95 | 0.4876 |
| 6 | DRB10403 | 1.15 | 0.2758 | 4.769 | 0.8505 |
| 6 | DRB10405 | 2.80 | 0.2454 | 32.04 | 0.4067 |
| 6 | DRB11104 | 1.61 | 0.3782 | 6.825 | 0.5206 |
| 6 | DRB10103 | 8.81E-09 | 0 | inf | 0.9983 |
| 6 | DRB10803 | 2.62E-07 | 0 | inf | 0.9983 |
| 6 | DRB10402 | 1.68E-06 | 0 | inf | 0.9992 |
| 6 | DRB11601 | 0.72 | 0.2851 | 1.819 | 0.4877 |
| 6 | DRB11602 | 7.30E-07 | 0 | inf | 0.9993 |
| 6 | DRB11103 | 3.83E-06 | 0 | inf | 0.9993 |
| 6 | DRB11502 | 1.03E-09 | 0 | inf | 0.9977 |
| 6 | DRB11102 | 1.74E-05 | 0 | inf | 0.9996 |
| 6 | DQA10101 | 2.10 | 1.46 | 2.994 | 5.62E-05 |
| 6 | DQA10102 | 1.34 | 1.014 | 1.775 | 0.03979 |
| 6 | DQA10501 | 1.33 | 0.812 | 2.162 | 0.2601 |
| 6 | DQA10104 | 1.45 | 0.5343 | 3.914 | 0.4677 |
| 6 | DQA10401 | 1.02 | 0.7326 | 1.425 | 0.8989 |
| 6 | DQA10103 | 0.88 | 0.6092 | 1.268 | 0.4896 |
| 6 | DQA10201 | 0.95 | 0.6229 | 1.448 | 0.8101 |
| 6 | DQA10303 | 0.46 | 0.2306 | 0.9186 | 0.02776 |
| 6 | DQA10302 | 0.93 | 0.5697 | 1.509 | 0.7614 |
| 6 | DQA10301 | 0.56 | 0.4004 | 0.7935 | 0.001017 |
| 6 | DQA10505 | 0.93 | 0.6476 | 1.342 | 0.7063 |
| 6 | DQA10105 | 0.15 | 0.01861 | 1.187 | 0.07212 |
| 6 | DQA10509 | 7.41E-09 | 0 | inf | 0.9982 |
| 6 | DQA10601 | 2.62E-07 | 0 | inf | 0.9983 |
| 6 | DQB10501 | 1.84 | 1.3 | 2.618 | 0.0006121 |
| 6 | DQB10602 | 1.41 | 1.049 | 1.894 | 0.02275 |
| 6 | DQB10201 | 1.33 | 0.812 | 2.162 | 0.2601 |
| 6 | DQB10302 | 0.52 | 0.3699 | 0.7401 | 0.0002512 |
| 6 | DQB10303 | 1.22 | 0.8108 | 1.836 | 0.3401 |
| 6 | DQB10301 | 0.76 | 0.5463 | 1.067 | 0.1135 |
| 6 | DQB10402 | 1.08 | 0.7745 | 1.496 | 0.6608 |
| 6 | DQB10202 | 0.66 | 0.3969 | 1.106 | 0.1152 |
| 6 | DQB10502 | 0.71 | 0.2916 | 1.732 | 0.4524 |
| 6 | DQB10603 | 0.86 | 0.5944 | 1.241 | 0.4176 |
| 6 | DQB10604 | 1.27 | 0.7517 | 2.152 | 0.37 |
| 6 | DQB10503 | 1.40 | 0.5059 | 3.868 | 0.5177 |
| 6 | DQB10504 | 5.78 | 0.5957 | 56.09 | 0.1302 |
| 6 | DQB10319 | 1.74E-05 | 0 | inf | 0.9996 |
| 6 | DQB10304 | 7.86E-07 | 0 | inf | 0.9986 |
| 6 | DQB10609 | 2.18 | 0.1954 | 24.4 | 0.5259 |
| 6 | DQB10601 | 6.31E-08 | 0 | inf | 0.9988 |
| 6 | A0301 | 1.20 | 0.8928 | 1.618 | 0.2253 |
| 6 | A0201 | 1.01 | 0.7426 | 1.382 | 0.9344 |
| 6 | A0101 | 0.97 | 0.5809 | 1.609 | 0.8969 |
| 6 | A1101 | 1.16 | 0.7434 | 1.815 | 0.5109 |
| 6 | A2402 | 0.87 | 0.6093 | 1.252 | 0.4605 |
| 6 | A3101 | 0.66 | 0.3545 | 1.215 | 0.1801 |
| 6 | A2501 | 0.69 | 0.3027 | 1.552 | 0.3648 |
| 6 | A6801 | 0.99 | 0.6551 | 1.482 | 0.9438 |
| 6 | A3002 | 0.55 | 0.0776 | 3.865 | 0.5459 |
| 6 | A2601 | 1.53 | 0.655 | 3.594 | 0.3243 |
| 6 | A2301 | 1.69 | 0.4494 | 6.324 | 0.4387 |
| 6 | A3201 | 0.52 | 0.2974 | 0.9094 | 0.02186 |
| 6 | A2901 | 2.16 | 0.488 | 9.549 | 0.3104 |
| 6 | A0205 | 2.94E-08 | 0 | inf | 0.9979 |
| 6 | A3303 | 6.41 | 0.9783 | 41.98 | 0.05274 |
| 6 | A2902 | 0.47 | 0.04527 | 4.884 | 0.5274 |
| 6 | A3001 | 1.46 | 0.3369 | 6.29 | 0.615 |
| 6 | A0206 | 4.15E-06 | 0 | inf | 0.9996 |
| 6 | A6802 | 87.1 | 5.807 | 1307 | 0.001225 |
| 6 | A6901 | 0.89 | 0.08727 | 9.075 | 0.9216 |
| 6 | A3301 | 4.11E-07 | 0 | inf | 0.9988 |
| 6 | A6601 | 6.18E-08 | 0 | inf | 0.9992 |
| 6 | A0202 | 3.48E-08 | 0 | inf | 0.9989 |
| 6 | A3402 | 6.50E-05 | 0 | inf | 0.9997 |
| 6 | A3004 | 1.74E-05 | 0 | inf | 0.9996 |
| 6 | C0202 | 1.37 | 0.9226 | 2.022 | 0.1193 |
| 6 | C0304 | 1.07 | 0.7669 | 1.485 | 0.7001 |
| 6 | C0501 | 0.69 | 0.4522 | 1.066 | 0.09557 |
| 6 | C0704 | 0.98 | 0.4962 | 1.917 | 0.9422 |
| 6 | C0401 | 1.04 | 0.698 | 1.554 | 0.8418 |
| 6 | C0701 | 1.04 | 0.6972 | 1.561 | 0.837 |
| 6 | C0102 | 1.21 | 0.8311 | 1.764 | 0.3192 |
| 6 | C0602 | 1.21 | 0.8206 | 1.79 | 0.3339 |
| 6 | C0702 | 0.93 | 0.6863 | 1.252 | 0.6208 |
| 6 | C0303 | 0.86 | 0.6022 | 1.222 | 0.3961 |
| 6 | C0310 | 2.62E+09 | 0 | inf | 0.9993 |
| 6 | C0302 | 2.77 | 0.5301 | 14.5 | 0.227 |
| 6 | C1502 | 1.13 | 0.6556 | 1.929 | 0.6696 |
| 6 | C1402 | 0.58 | 0.2423 | 1.368 | 0.2111 |
| 6 | C1203 | 0.75 | 0.4382 | 1.294 | 0.3048 |
| 6 | C0801 | 5.68E-08 | 0 | inf | 0.9986 |
| 6 | C0802 | 0.92 | 0.06755 | 12.48 | 0.9488 |
| 6 | C0803 | 5.50E-09 | 0 | inf | 0.9976 |
| 6 | C1701 | 0.77 | 0.2625 | 2.255 | 0.6325 |
| 6 | C1601 | 0.46 | 0.05021 | 4.197 | 0.4904 |
| 6 | C1202 | 7.80E-10 | 0 | inf | 0.9979 |
| 6 | C1403 | 1.37E+11 | 0 | inf | 0.9992 |
| 6 | C1602 | 6.80E-08 | 0 | inf | 0.9995 |
| 6 | C1505 | 8.68E-09 | 0 | inf | 0.9987 |
| 6 | B1501 | 0.85 | 0.6167 | 1.173 | 0.324 |
| 6 | B4001 | 0.98 | 0.6392 | 1.508 | 0.9328 |
| 6 | B0801 | 1.18 | 0.6764 | 2.063 | 0.5582 |
| 6 | B4402 | 0.82 | 0.5595 | 1.199 | 0.3039 |
| 6 | B0702 | 0.91 | 0.6685 | 1.247 | 0.5678 |
| 6 | B1801 | 0.58 | 0.369 | 0.91 | 0.01781 |
| 6 | B2705 | 1.49 | 1.041 | 2.13 | 0.02933 |
| 6 | B3501 | 1.17 | 0.7647 | 1.785 | 0.4719 |
| 6 | B1302 | 0.73 | 0.4082 | 1.31 | 0.2924 |
| 6 | B3901 | 0.70 | 0.3913 | 1.255 | 0.2317 |
| 6 | B4002 | 1.48 | 0.8843 | 2.489 | 0.1351 |
| 6 | B3503 | 1.69 | 0.5908 | 4.815 | 0.3288 |
| 6 | B4101 | 1.66 | 0.3866 | 7.121 | 0.4957 |
| 6 | B3924 | 15.5 | 2.607 | 92.36 | 0.002587 |
| 6 | B3906 | 1.34 | 0.2536 | 7.044 | 0.7322 |
| 6 | B1517 | 1.41 | 0.1451 | 13.62 | 0.7687 |
| 6 | B5101 | 0.95 | 0.6207 | 1.441 | 0.7953 |
| 6 | B3508 | 0.74 | 0.07694 | 7.141 | 0.7956 |
| 6 | B5701 | 1.16 | 0.5339 | 2.537 | 0.7026 |
| 6 | B5601 | 0.83 | 0.4064 | 1.707 | 0.6176 |
| 6 | B2702 | 0.82 | 0.07836 | 8.524 | 0.8661 |
| 6 | B4901 | 10.8 | 1.908 | 61.4 | 0.007149 |
| 6 | B4701 | 2.87 | 1.312 | 6.294 | 0.00833 |
| 6 | B3502 | 0.71 | 0.01008 | 50.63 | 0.877 |
| 6 | B3701 | 1.94 | 0.6974 | 5.405 | 0.204 |
| 6 | B1402 | 6.16E-08 | 0 | inf | 0.9967 |
| 6 | B4501 | 4.33E-08 | 0 | inf | 0.998 |
| 6 | B3801 | 1.27 | 0.491 | 3.295 | 0.6204 |
| 6 | B5201 | 7.80E-10 | 0 | inf | 0.9979 |
| 6 | B4801 | 8.31E-09 | 0 | inf | 0.997 |
| 6 | B1518 | 2.47E-08 | 0 | inf | 0.9983 |
| 6 | B5501 | 0.65 | 0.1328 | 3.229 | 0.6031 |
| 6 | B4102 | 0.22 | 0.02232 | 2.128 | 0.19 |
| 6 | B4403 | 1.49 | 0.4968 | 4.452 | 0.478 |
| 6 | B5001 | 4.25E-08 | 0 | inf | 0.9976 |
| 6 | B1401 | 4.34 | 0.05208 | 360.9 | 0.5156 |
| 6 | B0705 | 8.68E-09 | 0 | inf | 0.9987 |
| 6 | B4405 | 0.39 | 0.03412 | 4.421 | 0.446 |
| 6 | B5801 | 2.32 | 0.455 | 11.81 | 0.3115 |
| 6 | B5301 | 3.39E-08 | 0 | inf | 0.9994 |
| 6 | DPB10301 | 1.25 | 0.9335 | 1.673 | 0.1343 |
| 6 | DPB10101 | 1.27 | 0.7543 | 2.124 | 0.3723 |
| 6 | DPB10201 | 0.70 | 0.5118 | 0.956 | 0.02495 |
| 6 | DPB10402 | 1.09 | 0.8096 | 1.476 | 0.5613 |
| 6 | DPB10401 | 1.02 | 0.7702 | 1.347 | 0.8971 |
| 6 | DPB110401 | 1.77 | 0.565 | 5.567 | 0.3262 |
| 6 | DPB10501 | 1.18 | 0.6517 | 2.118 | 0.5919 |
| 6 | DPB11401 | 0.82 | 0.3393 | 1.97 | 0.6533 |
| 6 | DPB10901 | 1.40 | 0.239 | 8.138 | 0.7116 |
| 6 | DPB11501 | 0.30 | 0.03423 | 2.636 | 0.2778 |
| 6 | DPB11601 | 0.78 | 0.1475 | 4.151 | 0.7732 |
| 6 | DPB11901 | 2.17 | 0.4702 | 9.996 | 0.321 |
| 6 | DPB11101 | 4.43E-08 | 0 | inf | 0.9971 |
| 6 | DPB11001 | 1.24E-08 | 0 | inf | 0.9955 |
| 6 | DPB11701 | 0.37 | 0.03555 | 3.908 | 0.4105 |
| 6 | DPB11301 | 0.39 | 0.03356 | 4.56 | 0.4539 |
| 6 | DPB12301 | 1.21E-07 | 0 | inf | 0.999 |

*Supplementary Table 3. DRB1 alleles, amino acids and HLA-SNPs with*

P < 0.0001

| **CHR** | **SNP or Allele** | **BP** | **A1** | **OR** | **SE** | **L95** | **U95** | **P** |
| --- | --- | --- | --- | --- | --- | --- | --- | --- |
| 6 | rs9268528 | 32491086 | G | 0.58 | 0.1127 | 0.462 | 0.7185 | 9.89E-07 |
| 6 | rs9268542 | 32492699 | G | 0.58 | 0.1127 | 0.4633 | 0.7205 | 1.12E-06 |
| 6 | Position.11V | 32578807 | P | 0.49 | 0.1502 | 0.3666 | 0.6607 | 2.37E-06 |
| 6 | rs2239804 | 32519501 | G | 0.61 | 0.1117 | 0.4927 | 0.7633 | 1.19E-05 |
| 6 | Position.13H | 32578815 | P | 0.52 | 0.1509 | 0.387 | 0.6991 | 1.48E-05 |
| 6 | Position.33N | 32578842 | A | 0.52 | 0.1509 | 0.387 | 0.6991 | 1.48E-05 |
| 6 | Position.33H | 32578843 | P | 0.52 | 0.1509 | 0.387 | 0.6991 | 1.48E-05 |
| 6 | DRB10101 | 32578769 | P | 2.19 | 0.1836 | 1.53 | 3.141 | 1.91E-05 |
| 6 | Position.26T | 32637416 | A | 0.57 | 0.1344 | 0.4407 | 0.7464 | 3.52E-05 |
| 6 | Position.26S | 32637417 | P | 0.57 | 0.1344 | 0.4407 | 0.7464 | 3.52E-05 |
| 6 | Position.47Q | 32637428 | P | 0.57 | 0.1344 | 0.4407 | 0.7464 | 3.52E-05 |
| 6 | Position.56R | 32637449 | P | 0.57 | 0.1344 | 0.4407 | 0.7464 | 3.52E-05 |
| 6 | Position.76V | 32637463 | P | 0.57 | 0.1344 | 0.4407 | 0.7464 | 3.52E-05 |
| 6 | DQA10101 | 32637396 | P | 2.09 | 0.1831 | 1.46 | 2.994 | 5.62E-05 |
| 6 | rs2395163 | 32495787 | G | 0.59 | 0.1309 | 0.4581 | 0.7652 | 6.23E-05 |
| 6 | DRB10404 | 32578772 | P | 0.32 | 0.2928 | 0.18 | 0.5672 | 9.75E-05 |

Supplementary Table 4. The minor allele frequencies of the eight variants included in the replication analyses in HapMap-CEU, GWAS, and replication samples.

|  |  |  |  |  | **Minor allele frequencies (MAFs)** | | | | |
| --- | --- | --- | --- | --- | --- | --- | --- | --- | --- |
|  |  | **Gene (nearest gene)** |  |  | **HapMap-CEU** | **GWAS** |  | **REPL** |  |
| **SNP** | **BP (GRCh37)** |  | **A1** | **A2** |  | **Case** | **Control** | **Case** | **Control** |
| rs4714329 | 40273457 | (LINC00951) | G | A | 0.381 | 0.4432 | 0.3855 | 0.5087 | 0.3777 |
| rs9471290 | 40260515 | (LINC00951) | A | G | 0.35 | 0.4297 | 0.3667 | 0.4393 | 0.3571 |
| rs6458146 | 40218128 | (LINC00951) | G | A | 0.305 | 0.3932 | 0.327 | 0.3555 | 0.3172 |
| rs10498746 | 40224268 | (LINC00951) | G | A | 0.257 | 0.3527 | 0.2809 | 0.3035 | 0.2704 |
| rs2395163 | 32387809 | (HLA-DRA) | G | A | 0.274 | 0.1365 | 0.1903 | 0.1532 | 0.1883 |
| rs2239804 | 32411523 | HLA-DRA | G | A | 0.478 | 0.25 | 0.3219 | 0.3815 | 0.3147 |
| rs9268528 | 32383108 | (HLA-DRA) | G | A | 0.389 | 0.2486 | 0.3296 | 0.4017 | 0.3209 |
| rs9268542 | 32384721 | (HLA-DRA) | G | A | 0.397 | 0.25 | 0.3307 | 0.4104 | 0.3228 |
|  |  |  |  |  |  |  |  |  |  |

Supplementary Table 5 a. The linkage disequilibrium (LD) in the CRIME and the 1000 Genomes samples between rs1471329 and variants within the nearby genes (RP11-552E20.1, TDRG1, LINC00951, and LRFN2). The data is shown only for the variants with at least nominal LD (D’> 0.2, LOD > 2.0) in the CRIME sample.

| **Gene** | **SNP** | **BP(Hg18)** | **D'** | **LOD** | **r^2^** | **r^2^ in 1000G** |
| --- | --- | --- | --- | --- | --- | --- |
| LINC00951 | rs17619142 | 40420896 | 0.872 | 34.55 | 0.255 | 0.102 |
| LINC00951 | rs17619309 | 40427372 | 0.897 | 25.06 | 0.178 | 0.226 |
| TDRG1 | rs4714343 | 40452504 | 0.544 | 12.84 | 0.107 | 0.029 |
| TDRG1 | rs12664614 | 40453676 | 0.495 | 4.28 | 0.041 | 0.003 |
| TDRG1 | rs930250 | 40453891 | 0.262 | 4.11 | 0.042 | 0.04 |
| LRFN2 | rs930249 | 40471712 | 0.405 | 2.57 | 0.024 | 0 |
| LRFN2 | rs934465 | 40478477 | 0.215 | 3.25 | 0.033 | 0.004 |
| LRFN2 | rs6925172 | 40499542 | 0.570 | 4.03 | 0.035 | 0.027 |
| LRFN2 | rs13196792 | 40500347 | 0.373 | 14.21 | 0.129 | 0.009 |
| LRFN2 | rs12173741 | 40502153 | 0.299 | 5.21 | 0.051 | 0 |
| LRFN2 | rs10807240 | 40525174 | 0.317 | 3.75 | 0.035 | 0.017 |
| LRFN2 | rs2117154 | 40527857 | 0.317 | 3.75 | 0.035 | 0.017 |
| LRFN2 | rs1347257 | 40528210 | 0.231 | 3.16 | 0.028 | 0.007 |
| LRFN2 | rs2281263 | 40595767 | 0.399 | 4.73 | 0.047 | 0 |

Supplementary Table 5 b. The linkage disequilibrium (LD) in the HapMap3 and in the 1000 Genomes samples between rs4714329 and variants within the nearby genes (RP11-552E20.1, TDRG1, LINC00951, and LRFN2). The data is shown only for the variants with at least nominal LD (D’> 0.2, LOD > 2.0) in the HapMap3 sample.

| **Gene** | **SNP** | **BP(Hg18)** | **D'** | **LOD** | **r^2^** | **r^2^ in 1000G** |
| --- | --- | --- | --- | --- | --- | --- |
| RP11-552E20.1 | rs9394677 | 40348774 | 0.606 | 4.96 | 0.268 | 0.182 |
| LINC00951 | rs2504824 | 40421434 | 1 | 3.83 | 0.177 | 0.151 |
| LINC00951 | rs12206539 | 40428052 | 1 | 3.60 | 0.156 | 0.226 |
| LINC00951 | rs2477755 | 40424273 | 0.830 | 2.61 | 0.137 | 0.104 |
| LINC00951 | rs16868911 | 40425447 | 0.830 | 2.61 | 0.137 | 0.104 |
| LINC00951 | rs17619309 | 40427372 | 0.819 | 2.41 | 0.119 | 0.226 |
| LINC00951 | rs2477757 | 40429756 | 0.819 | 2.41 | 0.119 | 0.088 |
| LINC00951 | rs13199110 | 40421277 | 0.668 | 4.55 | 0.264 | 0.335 |
| LINC00951 | rs6458151 | 40431907 | 0.819 | 2.41 | 0.119 | 0.088 |
| LINC00951 | rs966082 | 40432272 | 0.830 | 2.61 | 0.137 | 0.104 |
| LINC00951 | rs17619142 | 40420896 | 0.545 | 3.32 | 0.109 | 0.102 |
| LINC00951 | rs13203076 | 40420324 | 0.590 | 2.37 | 0.114 | 0.102 |
| LINC00951 | rs9462610 | 40421580 | 0.545 | 2.32 | 0.109 | 0.102 |
| LINC00951 | rs17681038 | 40421908 | 0.545 | 2.32 | 0.109 | 0.102 |
| LINC00951 | rs17681074 | 40423283 | 0.562 | 2.03 | 0.106 | 0.088 |
| LINC00951 | rs730743 | 40424890 | 0.567 | 3.95 | 0.209 | 0.276 |

Supplementary Table 6 a. Results from the GTEx Portal investigation of rs4714329 association with TDRG1, LINC00951, and LRFN2 genes expression in brain and testis tissues. No data was available in GTEx Portal for RP11-552E20.1 gene expression. The most significant associations are bolded in the table (Adapted from GTEx Portal gene-association results tables for LINC00951, LRFN2 and TDRG1 genes.).

|  |  | **GENE** | | | | | |
| --- | --- | --- | --- | --- | --- | --- | --- |
|  |  | **LINC00951** | | **LRFN2** | | **TDRG1** | |
| **Tissue** | **N** | **P -Value** | **Effect Size** | **P-Value** | **Effect Size** | **P -Value** | **Effect Size** |
| Brain - Amygdala | 62 | 0.84 | 0.027 | 0.3 | -0.055 | 0.27 | -0.17 |
|
| Brain - Anterior cingulate cortex (BA24) | 72 | 0.35 | -0.12 | 0.6 | -0.035 | 0.19 | -0.17 |
|
| Brain - Caudate (basal ganglia) | 100 | 0.8 | 0.034 | 0.16 | -0.19 | 0.22 | 0.19 |
|
| Brain - Cerebellar Hemisphere | 89 | **0.0015** | **0.34** | **2E-05** | **0.56** | **4E-05** | **0.51** |
|
| Brain - Cerebellum | 103 | **2E-06** | **0.51** | **0.0002** | **0.48** | **0.0012** | **0.37** |
|
| Brain - Cortex | 96 | 0.53 | -0.059 | 0.41 | 0.051 | 0.23 | -0.12 |
|
| Brain - Frontal Cortex (BA9) | 92 | 0.4 | 0.072 | 0.62 | 0.031 | 0.54 | 0.064 |
|
| Brain - Hippocampus | 81 | 0.99 | 0.0012 | 0.14 | 0.085 | 0.48 | 0.091 |
|
| Brain - Hypothalamus | 81 | 0.61 | 0.066 | 0.84 | 0.018 | 0.22 | -0.18 |
|
| Brain - Nucleus accumbens (basal ganglia) | 72 | 0.49 | -0.083 | 0.54 | 0.057 | 0.36 | -0.11 |
| Brain - Putamen (basal ganglia) | 82 | 1 | 0.00034 | 0.35 | 0.15 | 0.4 | 0.13 |
|
| Brain - Spinal cord (cervical c-1) | 59 | 0.11 | -0.19 | 0.3 | 0.084 | 0.52 | 0.096 |
|
| Brain - Substantia nigra | 56 | 0.87 | 0.029 | 0.4 | -0.082 | 0.49 | 0.12 |
|
| Testis | 157 | 0.76 | 0.032 | 0.22 | -0.12 | 0.84 | -0.012 |
|
|  |  |  |  |  |  |  |  |

*Supplementary Table 6 b. The ten most significant associations from the Braineac database investigation of rs4714329 association with gene expression in ten brain tissues. No data was available for LINC00951 and RP11-552E20.1 gene expression. The most significant association is bolded in the table. (Adapted from Braineac Cis-eQTL results table.)*

|  | **geneSymbol** | **exprID** | **aveALL** | | **CRBL** | **FCTX** | **HIPP** | **MEDU** | **OCTX** | **PUTM** | **SNIG** | **TCTX** | **THAL** | **WHMT** |
| --- | --- | --- | --- | --- | --- | --- | --- | --- | --- | --- | --- | --- | --- | --- |
|  | **LRFN2** | t2953287 | 0.37 | | **0.00074** | 0.21 | 0.18 | 0.17 | 0.98 | 0.44 | 0.71 | 0.91 | 0.19 | 0.79 |
|  | LRFN2 | 2953303 | 0.18 | | 0.00099 | 0.78 | 0.39 | 0.13 | 0.90 | 0.40 | 0.81 | 0.72 | 0.29 | 0.47 |
|  | KIF6 | 2953083 | 0.93 | | 0.26 | 0.91 | 0.047 | 0.83 | 0.97 | 0.0017 | 0.84 | 0.32 | 0.91 | 0.27 |
|  | KIF6 | t2953082 | 0.93 | | 0.26 | 0.91 | 0.047 | 0.83 | 0.97 | 0.0017 | 0.84 | 0.32 | 0.91 | 0.27 |
|  | LRFN2 | 2953289 | 0.53 | | 0.0017 | 0.08 | 0.90 | 0.08 | 0.93 | 0.69 | 0.7 | 0.67 | 0.063 | 0.49 |
|  | NFYA,LOC221442 | 2906679 | 0.16 | | 0.52 | 0.71 | 0.93 | 0.55 | 0.0018 | 0.71 | 0.25 | 0.39 | 0.70 | 0.69 |
|  | FLJ41649 | 2953266 | 0.0087 | | 0.052 | 0.067 | 0.92 | 0.81 | 0.0029 | 0.36 | 0.4 | 0.83 | 0.93 | 0.96 |
|  | C6orf130,UNC5CL | 2953446 | 0.23 | | 0.19 | 0.36 | 0.62 | 0.29 | 0.0033 | 0.71 | 0.77 | 0.97 | 0.84 | 0.099 |
|  | NFYA,LOC221442 | 2906717 | 0.46 | | 0.85 | 0.85 | 0.93 | 0.79 | 0.017 | 0.41 | 0.83 | 0.0036 | 0.39 | 0.59 |
|  | TDRG1 | 2906482 | 0.28 | | 0.13 | 0.66 | 0.71 | 0.65 | 0.0038 | 0.089 | 0.51 | 0.35 | 0.91 | 0.23 |
| CRBL = cerebellar cortex | | | |  | | | | | | | | | | |
| FCTX = frontal cortex | | | |  | | | | | | | | | | |
| HIPP = hippocampus | | | |  | | | | | | | | | | |
| MEDU = medulla | | | |  | | | | | | | | | | |
| OCTX = occipital cortex | | | |  | | | | | | | | | | |
| PUTM = putamen | | | |  | | | | | | | | | | |
| SNIG = substantia nigra | | | |  | | | | | | | | | | |
| TCTX = temporal cortex | | | |  | | | | | | | | | | |
| THAL = thalamus | | | |  | | | | | | | | | | |
| WHMT = white matter | | | |  | | | | | | | | | | |

Supplementary Table 7. SCID-II questions and the corresponding item numbers and SCID-II question numbers.

|  | **SCID-II QUESTION NUMBER** | **SCID-II QUESTION** |
| --- | --- | --- |
| 1 | 123 | “Often bullied, threatened, or intimidated others” |
| 2 | 124 | “Often initiated physical fights” |
| 3 | 125 | “Has used a weapon that can cause serious physical harm to others (e.g., a bat, brick, broken bottle, knife, gun)” |
| 4 | 126 | “Has been physically cruel to people” |
| 5 | 127 | “Has been physically cruel to animals” |
| 6 | 128 | “Has stolen while confronting a victim (e.g., mugging, purse snatching, extortion, armed robbery)” |
| 7 | 129 | “Has forced someone into sexual activity” |
| 8 | 130 | “Has deliberately engaged in fire setting with the intention of causing serious damage” |
| 9 | 131 | “Has deliberately destroyed other’s property (other than by fire setting)” |
| 10 | 132 | “Has broken into someone else’s house, building, or car” |
| 11 | 133 | “Often lies to obtain goods or favors or to avoid oblications (i.e., “cons” others)” |
| 12 | 134 | “Has stolen items of nontrivial value without confronting victim (e.g., shoplifting, stealing but without breaking and entering, forgery)” |
| 13 | 135 | “Has run away from home overnight at least twice while living in parental or parental surrogate home (or once without returning for a lengthy period)” |
| 14 | 136 | “Often stayed out at night despite parental prohibitions” |
| 15 | 137 | “Often truant from school” |
| 16 | 139 | “Failure to conform to social norms with respect to lawful behaviors, as indicated by repeatedly performing acts that are ground for arrest” |
| 17 | 140 | “Deceitfulness, as indicated by repeated lying, use of aliases, or conning others for personal profit or pleasure” |
| 18 | 141 | “Impulsivity or failure to plan ahead” |
| 19 | 142 | “Irritability and aggressiveness, as indicated by repeated physical fights or assaults” |
| 20 | 143 | “Reckless disregard for safety of self or others” |
| 21 | 144 | “Consistent irresponsibility, as indicated by repeated failure to sustain consistent work behavior or honor financial obligations” |
| 22 | 145 | “Lacks remorse as indicated by being indifferent to, or rationalizing having hurt, mistreated or stolen from another” |

*Supplementary Table 8. Results for the 22 individual SCID-II items. Item 7 is indicated with asterix and red colour for the inadequate number of participants in that specific item.*

|  | **GWAS** |  |  |  |  |  |  |  | |
| --- | --- | --- | --- | --- | --- | --- | --- | --- | --- |
| ITEM | N | OR | SE | L95 | U95 | P |  |  | |
| **1** | **208** | 1.635 | 0.1278 | 1.273 | 2.101 | 0.000119 |  |  | |
| **2** | **187** | 1.601 | 0.1327 | 1.234 | 2.077 | 0.00039 |  |  | |
| **3** | **166** | 1.551 | 0.1356 | 1.189 | 2.023 | 0.001211 |  |  | |
| **4** | **95** | 1.514 | 0.1714 | 1.082 | 2.118 | 0.01552 |  |  | |
| **5** | **69** | 1.479 | 0.194 | 1.011 | 2.162 | 0.04379 |  |  | |
| **6** | **101** | 1.48 | 0.1709 | 1.058 | 2.069 | 0.02189 |  |  | |
| **7*** | **2** | 1650000 | 174.3 | 7.6E-143 | 3.6E+154 | 0.9345 |  |  | |
| **8** | **64** | 1.537 | 0.217 | 1.004 | 2.351 | 0.0477 |  |  | |
| **9** | **163** | 1.57 | 0.142 | 1.189 | 2.074 | 0.001491 |  |  | |
| **10** | **253** | 1.486 | 0.1156 | 1.185 | 1.864 | 0.000608 |  |  | |
| **11** | **219** | 1.383 | 0.119 | 1.096 | 1.746 | 0.006392 |  |  | |
| **12** | **321** | 1.547 | 0.1034 | 1.263 | 1.894 | 2.46E-05 |  |  | |
| **13** | **227** | 1.537 | 0.1178 | 1.22 | 1.936 | 0.000263 |  |  | |
| **14** | **189** | 1.62 | 0.1267 | 1.264 | 2.077 | 0.000139 |  |  | |
| **15** | **178** | 1.422 | 0.1258 | 1.111 | 1.82 | 0.005139 |  |  | |
| **16** | **363** | 1.52 | 0.09808 | 1.254 | 1.842 | 1.99E-05 |  |  | |
| **17** | **116** | 1.516 | 0.1522 | 1.125 | 2.043 | 0.006253 |  |  | |
| **18** | **309** | 1.497 | 0.1057 | 1.217 | 1.841 | 0.000135 |  |  | |
| **19** | **306** | 1.451 | 0.1047 | 1.182 | 1.781 | 0.000379 |  |  | |
| **20** | **328** | 1.528 | 0.1015 | 1.253 | 1.864 | 2.93E-05 |  |  | |
| **21** | **283** | 1.547 | 0.1066 | 1.256 | 1.907 | 4.23E-05 |  |  | |
| **22** | **105** | 1.434 | 0.1561 | 1.056 | 1.947 | 0.02105 |  |  | |
|  |  |  |  |  |  |  |  |  | |
|  | **REPL** |  |  |  |  |  |  |  | |
| ITEM | N | OR | SE | L95 | U95 | P |  |  | |
| **1** | **82** | 1.617 | 0.1747 | 1.148 | 2.278 | 0.005919 |  |  | |
| **2** | **81** | 1.829 | 0.1843 | 1.274 | 2.625 | 0.001056 |  |  | |
| **3** | **61** | 1.723 | 0.2083 | 1.145 | 2.591 | 0.009032 |  |  | |
| **4** | **30** | 1.898 | 0.2843 | 1.087 | 3.314 | 0.02418 |  |  | |
| **5** | **15** | 1.108 | 0.3853 | 0.5205 | 2.357 | 0.7907 |  |  | |
| **6** | **54** | 2.168 | 0.2231 | 1.4 | 3.357 | 0.000525 |  |  | |
| **7*** | **1** | 1.699 | 1.448 | 0.09942 | 29.03 | 0.7144 |  |  | |
| **8** | **22** | 1.273 | 0.3172 | 0.6836 | 2.37 | 0.4467 |  |  | |
| **9** | **49** | 1.496 | 0.221 | 0.9698 | 2.306 | 0.06855 |  |  | |
| **10** | **102** | 1.706 | 0.1677 | 1.228 | 2.369 | 0.00145 |  |  | |
| **11** | **110** | 1.839 | 0.1549 | 1.358 | 2.491 | 8.39E-05 |  |  | |
| **12** | **143** | 1.553 | 0.1385 | 1.184 | 2.038 | 0.001476 |  |  | |
| **13** | **92** | 1.692 | 0.1675 | 1.218 | 2.349 | 0.001695 |  |  | |
| **14** | **81** | 1.71 | 0.1788 | 1.204 | 2.427 | 0.002694 |  |  | |
| **15** | **91** | 1.838 | 0.1653 | 1.329 | 2.541 | 0.000231 |  |  | |
| **16** | **166** | 1.755 | 0.1294 | 1.362 | 2.262 | 1.36E-05 |  |  | |
| **17** | **64** | 1.925 | 0.1946 | 1.314 | 2.818 | 0.000766 |  |  | |
| **18** | **132** | 1.536 | 0.1415 | 1.164 | 2.027 | 0.00243 |  |  | |
| **19** | **140** | 1.776 | 0.1391 | 1.352 | 2.332 | 3.65E-05 |  |  | |
| **20** | **149** | 1.919 | 0.137 | 1.467 | 2.51 | 1.95E-06 |  |  | |
| **21** | **138** | 1.903 | 0.1395 | 1.448 | 2.502 | 3.96E-06 |  |  | |
| **22** | **39** | 1.488 | 0.2404 | 0.9291 | 2.384 | 0.09811 |  |  | |
|  |  |  |  |  |  |  |  |  | |
|  | **META** |  |  |  |  |  |  |  | |
| ITEM | **N** | OR | OR_se | OR_95L | OR_95U | p-value | _-log10_p-value | | i2 |
| **1** | **290** | 1.628713 | 0.152044 | 1.330707 | 1.993456 | 2.27E-06 | 5.644153 | | 0 |
| **2** | **268** | 1.675458 | 0.162814 | 1.356343 | 2.069653 | 1.72E-06 | 5.76474 | | 0 |
| **3** | **227** | 1.600253 | 0.163072 | 1.280631 | 1.999646 | 3.58E-05 | 4.446696 | | 0 |
| **4** | **125** | 1.607998 | 0.205127 | 1.205948 | 2.144086 | 0.00122 | 2.913783 | | 0 |
| **5** | **84** | 1.39508 | 0.205045 | 0.993192 | 1.959589 | 0.054797 | 1.261244 | | 0 |
| **6** | **155** | 1.704999 | 0.203356 | 1.306421 | 2.225181 | 8.67E-05 | 4.062168 | | 0.45716 |
| **7*** | **3** | 1.700618 | 0.816885 | 0.099524 | 29.05922 | 0.713876 | 0.146377 | | 0 |
| **8** | **86** | 1.447239 | 0.21875 | 1.01849 | 2.056478 | 0.039207 | 1.406634 | | 0 |
| **9** | **212** | 1.548067 | 0.16478 | 1.225098 | 1.956178 | 0.000254 | 3.595961 | | 0 |
| **10** | **355** | 1.553461 | 0.134807 | 1.289239 | 1.871834 | 3.7E-06 | 5.432106 | | 0 |
| **11** | **329** | 1.53698 | 0.132147 | 1.277971 | 1.848483 | 5.07E-06 | 5.295005 | | 0.531888 |
| **12** | **464** | 1.549149 | 0.118508 | 1.316873 | 1.822395 | 1.31E-07 | 6.882278 | | 0 |
| **13** | **319** | 1.586599 | 0.139396 | 1.313383 | 1.916651 | 1.72E-06 | 5.76459 | | 0 |
| **14** | **270** | 1.649471 | 0.154334 | 1.346975 | 2.019899 | 1.31E-06 | 5.88279 | | 0 |
| **15** | **269** | 1.56236 | 0.142132 | 1.283782 | 1.901388 | 8.58E-06 | 5.066596 | | 0.343598 |
| **16** | **529** | 1.60198 | 0.116129 | 1.374368 | 1.867287 | 1.72E-09 | 8.765027 | | 0 |
| **17** | **180** | 1.659628 | 0.177381 | 1.311962 | 2.099425 | 2.43E-05 | 4.614512 | | 0 |
| **18** | **441** | 1.510846 | 0.117852 | 1.279856 | 1.783525 | 1.11E-06 | 5.955235 | | 0 |
| **19** | **446** | 1.560838 | 0.120388 | 1.324877 | 1.838823 | 1.04E-07 | 6.984293 | | 0.257913 |
| **20** | **477** | 1.656 | 0.12463 | 1.411725 | 1.942541 | 6.02E-10 | 9.220561 | | 0.440866 |
| **21** | **421** | 1.669424 | 0.130061 | 1.414505 | 1.970284 | 1.38E-09 | 8.85873 | | 0.283397 |
| **22** | **144** | 1.44982 | 0.167403 | 1.12171 | 1.873903 | 0.004562 | 2.340818 | | 0 |
|  |  |  |  |  |  |  |  |  | |

**Supplementary Figures**

*Supplementary Figure 1 a. Q-Q Plot of the association analysis of the combined sample of males and females.*

**A**

*Supplementary Figure 1 b. Q-Q Plot of the association analysis of the male sub-sample.*

**B**

***Supplementary Figure 2 a. Regional Manhattan Plot of chromosome 6 of the analysis of the combined sample of males and females.***

**A**

**
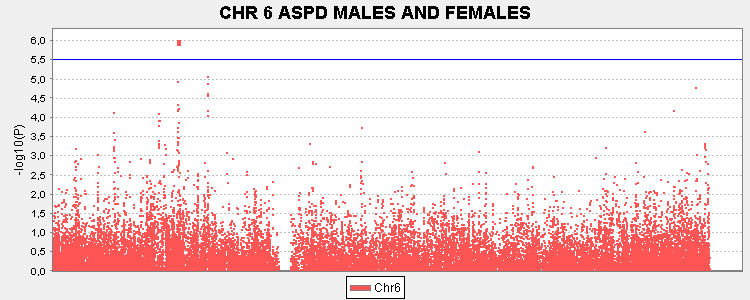
**

***Supplementary Figure 2 b. Regional Manhattan Plot of chromosome 6 of the male sub-sample.***

**B**


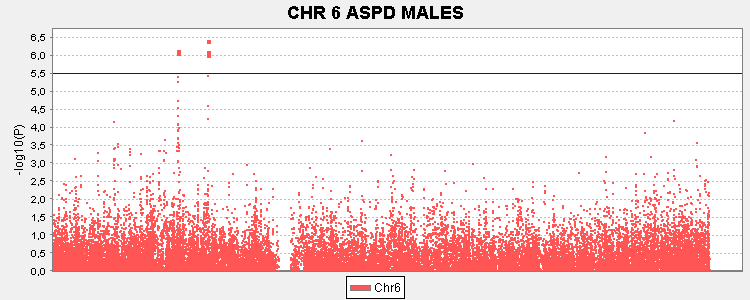


*Supplementary Figure 3. The Linkage Disequilibrium (LD) structure of the CRIME sample for rs4714329 and the other three selected CHR 6 SNPs on 6p21.2, created with Haploview.*


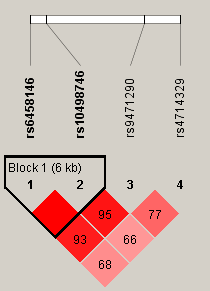


*Supplementary Figure 4 a. Rs4714329 linkage disequilibrium (LD) with the two SNPs of LINC00951 gene available in the CRIME sample, created with Haploview.*

**A**


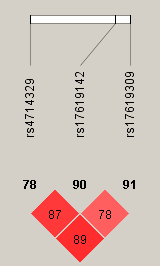


*Supplementary Figure 4 b. Rs4714329 linkage disequilibrium (LD) with the 23 SNPs of LINC00951 gene SNPs available in the HapMap3 sample, created with Haploview.*

**B**


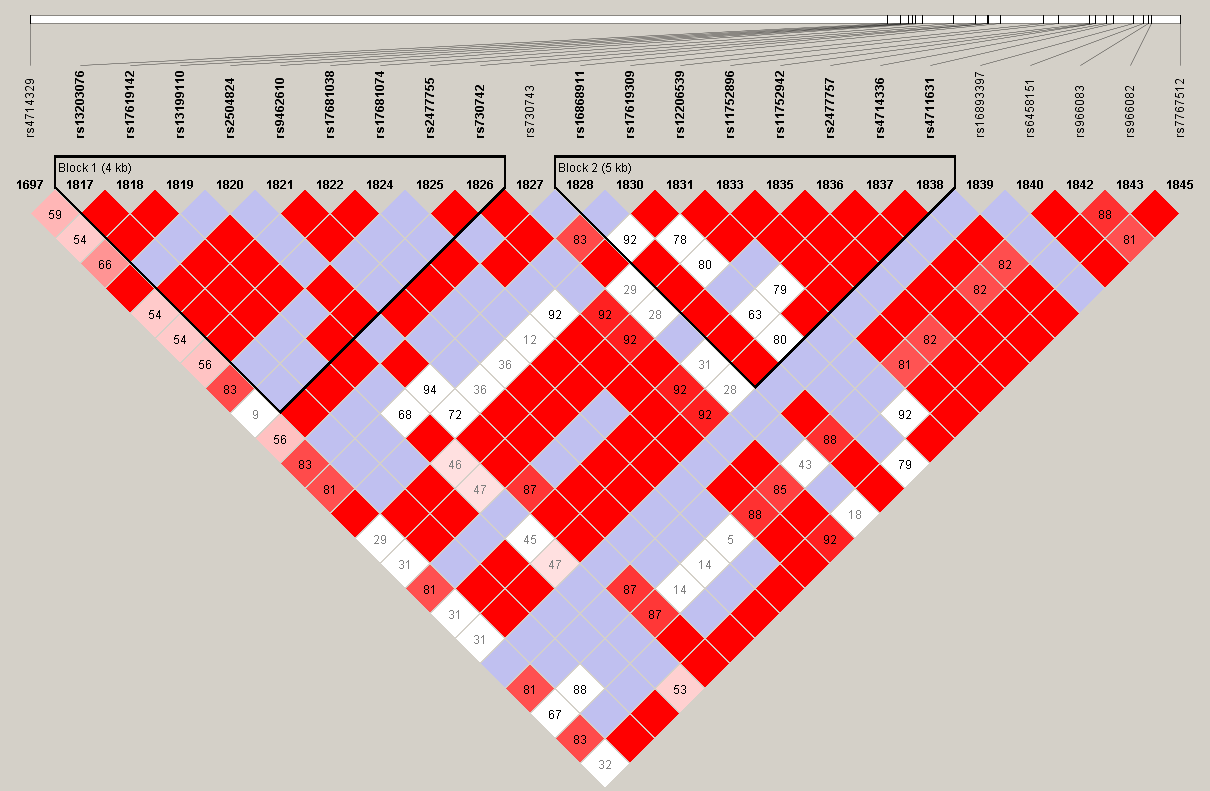


*Supplementary Figure 5.* *Results from the analysis of the 22 individual SCID-II items.*


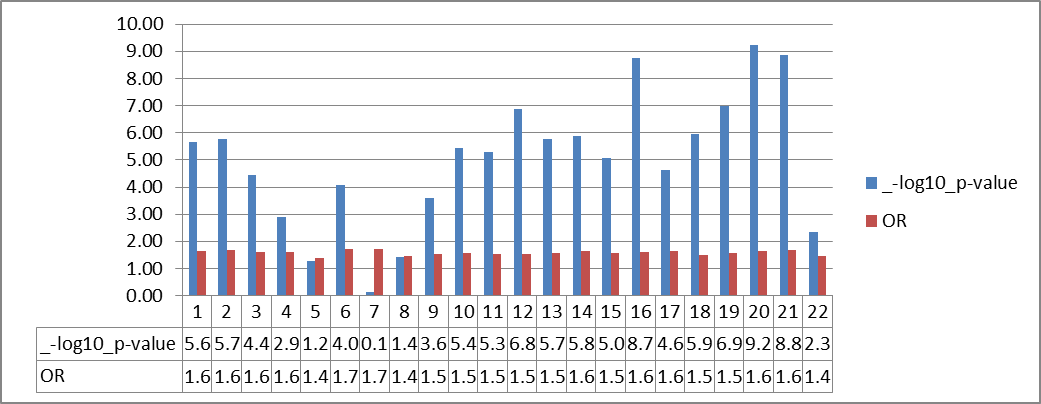

Supplement: Supplementary Informations [file tp2016155x1.doc]
